# A new megaraptoran dinosaur from the Lower Cretaceous Wessex Formation, UK, and its implications for theropod evolution and biogeography

**DOI:** 10.1098/rsos.260937

**Published:** 2026-10-14

**Authors:** Cassius Morrison, Charlie R. Scherer, Philip Mannion, Laura B. Porro, Brett Clark, Daniel D. Folkes, Paul M. Barrett

**Affiliations:** ^1^ Department of Earth Sciences, University College London, London, UK; ^2^ Fossil Reptiles, Amphibians and Birds Section, Natural History Museum, London, UK; ^3^ Cell and Developmental Biology, University College London Division of Biosciences, London, UK; ^4^ British Museum, London, UK; ^5^ School of Earth Sciences, University of Bristol, Bristol, UK; ^6^ University of the Witwatersrand Johannesburg Evolutionary Studies Institute, Johannesburg, South Africa

**Keywords:** megaraptora, endocast, Early Cretaceous, theropod, dinosaur, biogeography, Wessex Formation

## Abstract

Theropod dinosaur remains from the Barremian (Lower Cretaceous) Wessex Formation of the Isle of Wight, UK, are rare and often fragmentary. Here, we present a previously undescribed braincase from this formation that represents a new coelurosaurian, *Invictibellatrix amberina* gen. et sp. nov. Phylogenetic analysis and comparative anatomy indicate that *Invictibellatrix* is distantly related to all previously named theropods from this stratigraphic unit. Theropod braincases are often regarded as morphologically and phylogenetically conservative, but we demonstrate that neurocranial characters provide essential data that can help clarify the phylogenetic relationships of theropods. The sub-circular occipital condyle, sub-oval foramen magnum and presence of a sagittal nuchal crest indicate that *Invictibellatrix* is referable to Megaraptora, with phylogenetic analyses recovering it as an early-diverging member of this clade, within a paraphyletic array of Eurasian taxa. Incorporation of *Invictibellatrix* into a biogeographical analysis suggests that non-megaraptorid megaraptorans likely had a cosmopolitan distribution in the Early Cretaceous, and that the absence of megaraptorans in the Late Cretaceous of Laurasia is best explained by regional extinction. The recognition of this new genus increases our knowledge of Early Cretaceous European coelurosaurian diversity, which now includes tyrannosauroids, maniraptoriforms and megaraptorans.

## 1. Introduction

The Barremian (Lower Cretaceous) Wessex Formation crops out on the Isle of Wight, southern England, UK, and is part of the Wealden Supergroup [1]. Similarly aged ‘Wealden facies’ are widespread throughout Western Europe and have yielded abundant vertebrate remains that provide valuable information on dinosaurs during this interval. These assemblages consist of ornithopod and ankylosaurian ornithischians, titanosauriform and rebbachisaurid sauropods and various theropods, including spinosaurids, carcharodontosaurians and tyrannosauroids, among others (e.g. [2–8]).

Theropod remains have been collected from the Wessex Formation since the mid-nineteenth century, but these are often fragmentary, making it difficult to determine the taxonomic status and phylogenetic placement of this material. Theropod genera currently considered to be valid include the compsognathid *Aristosuchus* [9], the dromaeosaurids *Ornithodesmus* [10] and *Vectiraptor* [6], the carcharodontosaurid *Neovenator* [11], the spinosaurids *Ceratosuchops* and *Riparovenator* [12], the tyrannosauroid *Eotyrannus* [13] and the possible maniraptoran *Yaverlandia* ([14,15]; see also [16])*.* Two other taxa, *Calamospondylus* [9] and *Thecocoelurus* [7], might be referable to Oviraptorosauria and Ornithomimosauria, respectively, but their phylogenetic status is uncertain [17,18]. More broadly, within the Lower Cretaceous Wealden Supergroup, additional theropod genera include the spinosaurid *Baryonyx* [19], a possible carcharodontosaurian that is attributable to either *Altispinax* or *Becklespinax* (Maisch [20] regarding the taxonomy of this material), and the ornithomimosaurian *Valdoraptor* [21] (a potential junior synonym of *Thecocoelurus*: [17]). In addition, other material has been assigned to an indeterminate maniraptoran [22]. Excluding teeth, cranial material from Wealden theropods is limited to the partial skulls of *Neovenator* [11,23], *Eotyrannus* [13,24], *Ceratosuchops* [12], *Riparovenator* [12], *Baryonyx* [19,25] and *Yaverlandia* [15], with braincase material restricted to the three spinosaurids and the partial skull roof of *Yaverlandia* (table 1).

**Table 1.** Non-avian theropod taxa preserving cranial material (excluding teeth) from the wealden group.

genus	clade	stratigraphic age	preserved cranial material	source
*Neovenator*	Carcharodontosauria	Wessex Formation: Lower Cretaceous (Barremian)	left and right premaxillae, left maxilla, right nasal, right palatine, left dentary	Brusatte *et al.* [23]
*Eotyrannus*	Tyrannosauroidea	Wessex Formation: Lower Cretaceous (Barremian)	premaxilla, maxilla, nasal, lacrimal, prefrontal?, jugal, palatine, vomer?, quadrate, dentary, surangular	Naish & Cau [24]
*Ceratosuchops*	Spinosauridae	Wessex Formation: Lower Cretaceous (Barremian)	premaxilla, a near complete but disarticulated braincase. Referred material: right postorbital	Barker *et al.* [12]
*Riparovenator*	Spinosauridae	Wessex Formation: Lower Cretaceous (Barremian)	premaxilla, a disarticulated braincase, a left ‘preorbital’ fragment (partial lacrimal and prefrontal). Referred material: a posterior nasal fragment	Barker *et al.* [12]
*Baryonyx*	Spinosauridae	Weald Clay Formation: Lower Cretaceous (Barremian)	premaxilla, cojoined vomers, maxilla, nasals, lacrimal, prefrontal, postorbital, anterior end of braincase, jugal, quadrates, dentary, splenial, surangular, angular, coronoid	Charig & Milner [25]
*Yaverlandia*	?Maniraptora	Wessex Formation: Lower Cretaceous (Barremian)	skull roof	Naish & Martill [15]; Naish & Sweetman [16]

As Wealden Supergroup theropod remains have not always been studied in detail, it is possible that some of the unnamed specimens present in museum collections might represent additional taxa that would increase the diversity of this fauna, especially with respect to small- and medium-sized taxa, which are more difficult to find and less likely to be preserved (e.g. [26]). Conversely, it is possible that several of the named taxa should be synonymized, but these competing diversity signals are difficult to evaluate in the absence of foundational descriptive data and the often-limited anatomical overlap between the available specimens.

The specimen that forms the focus of this study, NHMUK PV R6775, is a partial braincase recovered from the foot of the cliff at Sudmoor Point, near Brook, on the southwest coast of the Isle of Wight. It was purchased by the NHMUK from Rev. R. W. Hooley in May 1924. It is from the Barremian-aged strata of the Wessex Formation and has never been formally described. Here, we provide three-dimensional reconstructions of the braincase and endocast based on µCT scans and describe its osteology and neurology. We make anatomical comparisons with other theropod braincases from the Wessex Formation and elsewhere and incorporate the specimen into two phylogenetic analyses. The latter are then used to quantitatively evaluate the biogeographic history of the taxon represented by NHMUK PV R6775 and its closest relatives.

### 1.1. Institutional abbreviations

NHMUK, Natural History Museum, London, UK; TMP, Royal Tyrell Museum, Drumheller, Canada.

### 1.2. Geological setting

The primary lithology of the Wessex Formation that outcrops on the Isle of Wight is a purple-red mudstone interbedded with sandstones. Its depositional environment is interpreted as a floodplain within a narrow valley [4]. The palaeoclimate is considered to have been semi-arid, owing to the presence of pedogenic calcrete nodules within the mudstone, as well as the presence of xerophytic ferns and the conifer genera *Pseudofrenelopsis* and *Watsoniocladus*, which had xerophytic adaptations [1,4]. Plant debris beds are a common feature of the formation and are the horizons from which most of the vertebrate fossils have been recovered [27]. The presence of large amounts of wood and pyrite in these debris beds suggests that they were formed from flash-flood events infilling existing topographical features, such as oxbow lakes, with the periodic abundance of charcoal indicative of seasonal wildfires [27]. Although the fossils found in the Wessex Formation are often disarticulated, in most cases there is little evidence for transportation prior to deposition [1]. The precise horizon from which NHMUK PV R6775 was collected is unknown, but given that Sudmoor Point has outcrops of L1 to L5 plant debris beds (*sensu* [27]), it is most plausible that NHMUK PV R6775 is from this stratigraphic section.

## 2. Methods

### 2.1. Micro-CT scanning and reconstruction

NHMUK PV R6775 was µCT scanned at the Imaging and Analysis Centre of the NHMUK with a Nikon HMX ST 225 µCT system (Nikon Metrology) at 210 kV and 200 µA using a copper filter with an isometric voxel size of 0.0665 mm voxel^–1^. The tomographic reconstructions were performed using Nikon CT-agent software (Nikon Metrology) to produce a Tagged Image File Format (TIFF) image stack. CT scans were segmented in Avizo v. 2021.2 to separate out regions of interest and reconstruct the braincase and endocast. For NHMUK PV R6775, density thresholding was used to initially separate bone from matrix before manual segmentation that involved interpolation across no more than five slices, following Porro *et al.* [28], as the scans had a good contrast between the bone and matrix, in addition to having only gradual changes in morphology. The same method was used to create the virtual endocast by digitally ‘filling’ the endocranial cavity.

### 2.2. Phylogenetic dataset and analysis

Two separate data matrices [24,29] were used to perform phylogenetic analyses to determine the position of the new taxon. The Naish & Cau [24] dataset samples a broad array of neotheropods, with 86 operational taxonomic units (OTUs) scored for 1759 characters. The Aranciaga Rolando *et al.* [29] dataset was designed to assess megaraptoran relationships and consists of 58 OTUs scored for 356 characters. In addition to NHMUK PV R6775, we also incorporated the megaraptoran *Murusraptor barrosaensis*, from the Late Cretaceous of Argentina, into the Naish & Cau [24] data matrix, which was scored from the literature [30,31]. We also revised scores for the existing *Spinosaurus aegyptiacus* OTU based on personal observations of NHMUK PV R16425, with these changes documented in the supplementary material. Characters (C) 112 and 632 were also revised in the Naish & Cau [24] dataset (see supplementary material for new scores) and a new character (1760) was added:

C112. Foramen magnum, shape: sub-circular, with approximately equal dorsoventral height to mediolateral width (0); elliptical, taller dorsoventrally than mediolaterally wide (1); elliptical, wider mediolaterally than dorsoventrally high (2). Unordered.

C632. Occipital condyle, shape: sub-circular, dorsoventral height equal to or greater than mediolateral width (0); elliptical, mediolaterally broader than dorsoventrally tall (1).

C1760. Foramen magnum, mediolateral width to that of occipital condyle ratio (FOR): 0.50 or lower (0); 0.51–0.64 (1); 0.65 to 0.95 (2); greater than 0.95 (3). Unordered.

Other than the inclusion of the new taxon, no changes were made to the Aranciaga Rolando *et al.* [29] data matrix. Both data matrices were analysed under maximum parsimony in TNT v. 1.6 [32,33]. We followed the protocols of previous iterations of these data matrices, with all characters unordered for the Naish & Cau [24] data matrix and 35 characters (1, 3, 5, 12, 14, 16, 26, 68, 105, 147, 154, 157, 159, 168, 170, 178, 180, 193, 194, 204, 207, 216, 234, 240, 258, 266, 270, 309, 315, 316, 317, 322, 323, 324 and 338) treated as ordered for the Aranciaga Rolando *et al.* [29] data matrix. In both instances, we ran a ‘new technology search’, using the ‘stabilize consensus’ option, with sectorial searches, drift and tree fusing all selected. After five rounds of consensus stabilizing, the resultant trees were used as the starting topologies for a ‘traditional search’, using tree bisection–reconnection. For both matrices, two versions of the analysis were run: one in which equal character weighting (EQW) was applied and a second, in which extended implied weighting (EIW) was implemented [34] using a *k*-value of nine following previous work (e.g. [35]).

To further elucidate the position of the new taxon within Megaraptora, we also ran a fossilized birth–death (FBD) analysis of the Aranciaga Rolando *et al.* [29] dataset in MrBayes v. 3.2.7a, following the methodology of Kotevski *et al.* [36]. This analysis was run for 20 000 000 generations and sampled every 2000. The first 25% were discarded as burn-in. Speciation, extinction and fossilization priors were set at exp (10), beta (1,1) and beta (1,1), respectively, and convergence was reached at an s.d. of split frequencies of <0.015. All nexus and TNT files are provided within the supplementary material.

### 2.3. Biogeographical analysis

We analysed the biogeography of Tyrannosauroidea, including Megaraptora, using both our EQW and EIW topologies derived from the Naish & Cau [24] data matrix, and performed ancestral area reconstructions using BioGeoBEARS [37] in R v. 4.3.1 [38]. We opted to use the results of our FBD analysis for the Aranciaga Rolando *et al.* [29] matrix because the increased resolution of this topology was necessary to conduct our subsequent biogeographical analyses, as BioGeoBEARS can only analyse historical biogeography using a fully bifurcating tree topology [37]. Eight biogeographical regions were considered here: Africa, Antarctica, Appalachia, Asia, Australia, Europe, Laramidia and South America as per [39]. Both topologies were calibrated using the cal3 method [40]. Time-stratified palaeogeographic constraints were used in the analyses to account for changes in connectivity between the biogeographical regions considered here. Connectivity between regions was assessed via the palaeogeographic reconstructions of the PALEOMAP project [41]. These dispersal constraints correspond to each stage of the latter half of the Mesozoic (Bajocian–Maastrichtian) and are provided in the supplementary material. Taxa in the EQW and EIW topologies generated here were assigned to one or more of our biogeographical regions, with each taxon occupying a maximum of three zones at any point in time. A preferred model was selected by comparing the sample-size corrected Akaike Information Criterion (AICc) and log likelihood (LnL) values of each model; the model with the lowest AICc and smallest LnL values was regarded as the best-fit model. BioGeoBEARS uses a file containing the geographic ranges of taxa to model different biogeographic processes. We followed the procedure of Mannion *et al.* [35] and Heath *et al.* [42] in incorporating a measure of sampling bias into our geographic range files, whereby the absence of an OTU from a spatiotemporal bin that lacks dinosaur-bearing deposits is scored as a ‘?’ rather than traditional binary coding (0 or 1). For example, Australia lacks latest Cretaceous terrestrial deposits [43], meaning that the absence of post-Turonian dinosaurs could be attributable to sampling bias rather than a genuine signal. Therefore, scoring taxa as ‘?’ allows us to account for this sampling heterogeneity. Similar biases occur in African and Antarctic strata, where sampling efforts are much less intensive compared with those in other regions (i.e. North America, South America, Europe and Asia). To account for sampling bias in these regions, all Late Cretaceous South American and Australian taxa are assigned a ‘?’ for Africa and Antarctica.

### 2.4. Plotting foramen magnum: occipital condyle ratios

We gathered data on the widths of the foramen magnum and occipital condyle from personal observations and the literature for as many non-maniraptoriform theropods as possible. We plotted the transverse foramen magnum width to transverse occipital condyle width ratio through time using an informal phylogeny of theropod relationships, using the stochastic character mapping algorithm in the ‘sim.map’ function of the phytools [44] R package.

## 3. Results

### 3.1. Systematic palaeontology

Dinosauria Owen, 1842

Saurischia Seeley, 1888

Theropoda Marsh, 1881

Tetanurae Gauthier, 1986

Coelurosauria Huene, 1914

Megaraptora [45]

*Invictibellatrix amberina* gen. et sp. nov.

Figures 1–3C

**Figure 1.**
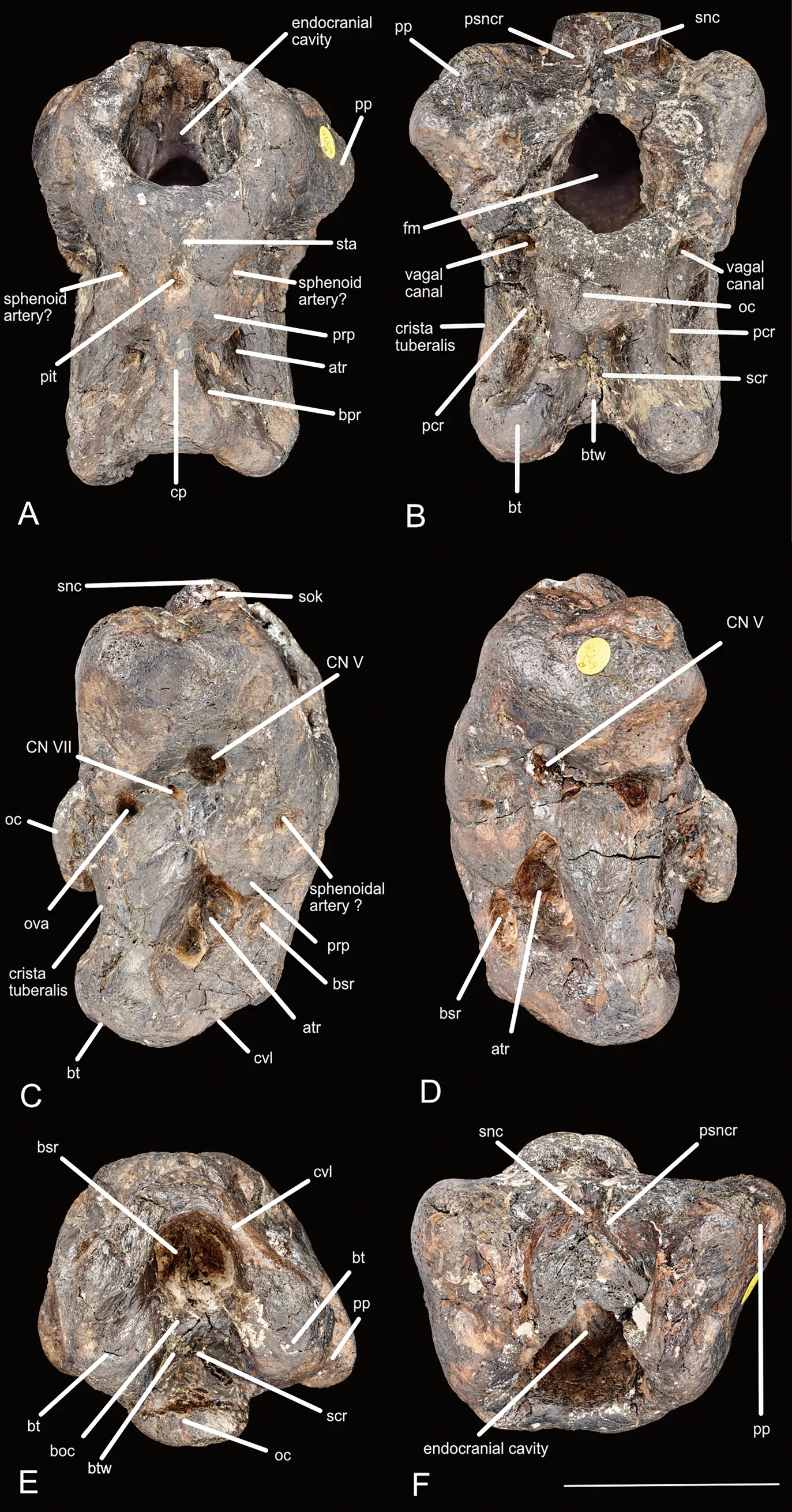
Photographs of the holotype braincase (NHMUK PV R6775) of *I. amberina* gen. et sp. nov. in A: anterior, B: posterior; C: right lateral; D: left lateral; E: ventral; F: dorsal views. Abbreviations: atr, anterior tympanic recess; boc, basioccipital crest; bpr, basipterygoid recess; bsr, basisphenoid recess; bt, basal tubera; btw, basituberal web; CN, cranial nerves V, VII; cp, cultriform process; cvl, crista ventrolateralis of basisphenoid; fm, foramen magnum; oc, occipital condyle; ova, fenestra ovalis; pcr, paracondylar recess; pit, depression of the pituitary gland within the pituitary fossa; pp, paraoccipital process; prp, preotic pendant; psncr, parasagittal nuchal crest recess; scr, sub-condylar recess; snc, parasagittal nuchal crest; sok, supraoccipital knob; sta, sella turcica. Scale bar equals 50 mm.

**Figure 2.**
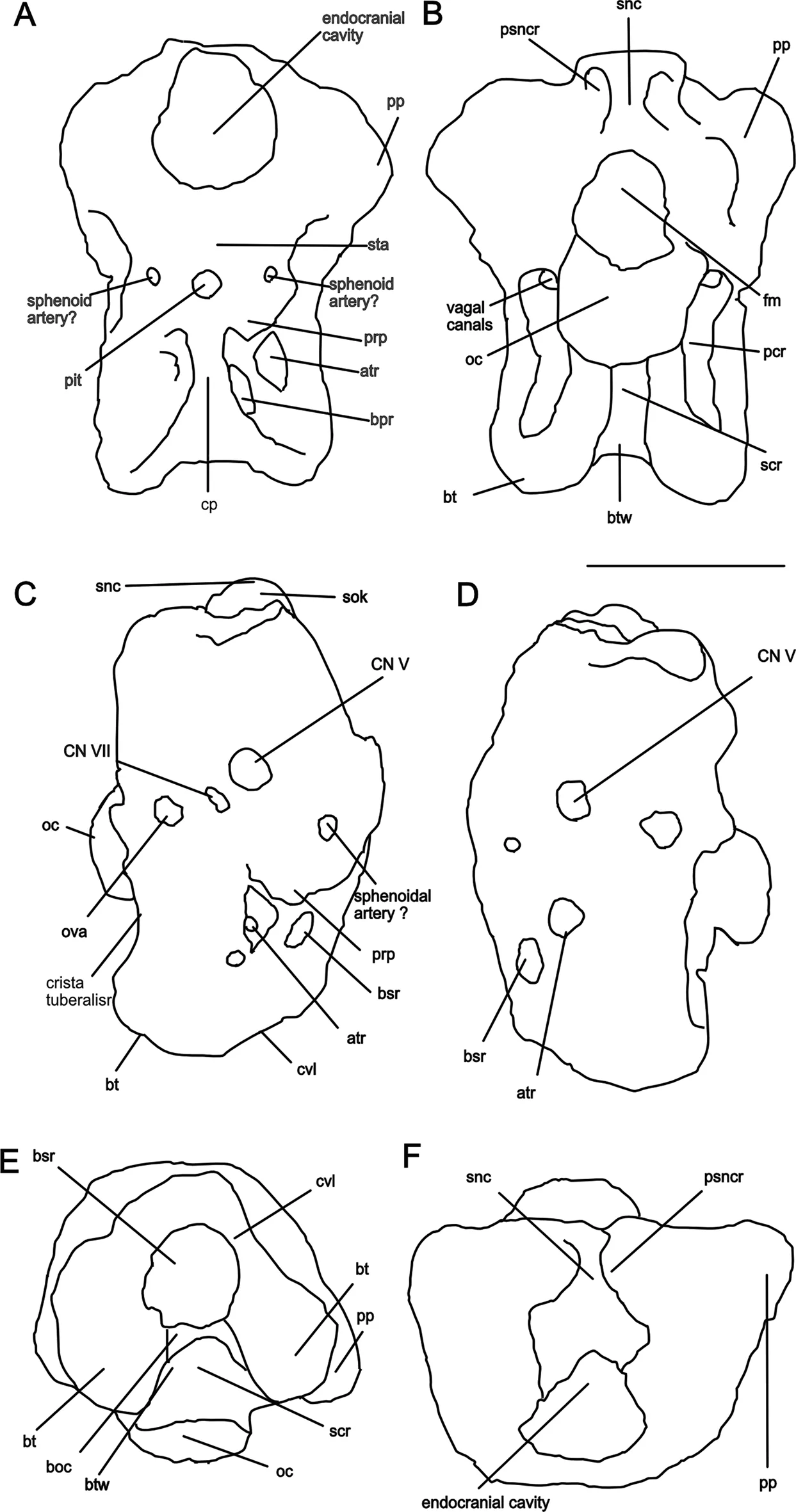
Line drawings of the holotype braincase (NHMUK PV R6775) of *I. amberina* gen. et sp. nov. in A: anterior, B: posterior; C: right lateral; D: left lateral; E: ventral; F: dorsal views. Abbreviations: atr, anterior tympanic recess; boc, basioccipital crest; bpr, basipterygoid recess; bsr, basisphenoid recess; bt, basal tubera; btw, basituberal web; CN, cranial nerves V, VII; cp, cultriform process; cvl, crista ventrolateralis of basisphenoid; fm, foramen magnum; oc, occipital condyle; ova, fenestra ovalis; pcr, paracondylar recess; pit, depression of the pituitary gland within the pituitary fossa; pp, paraoccipital process; prp, preotic pendant; psncr, parasagittal nuchal crest recess; scr, sub-condylar recess; snc, parasagittal nuchal crest; sok, supraoccipital knob; sta, sella turcica. Scale bar equals 50 mm.

**Figure 3.**
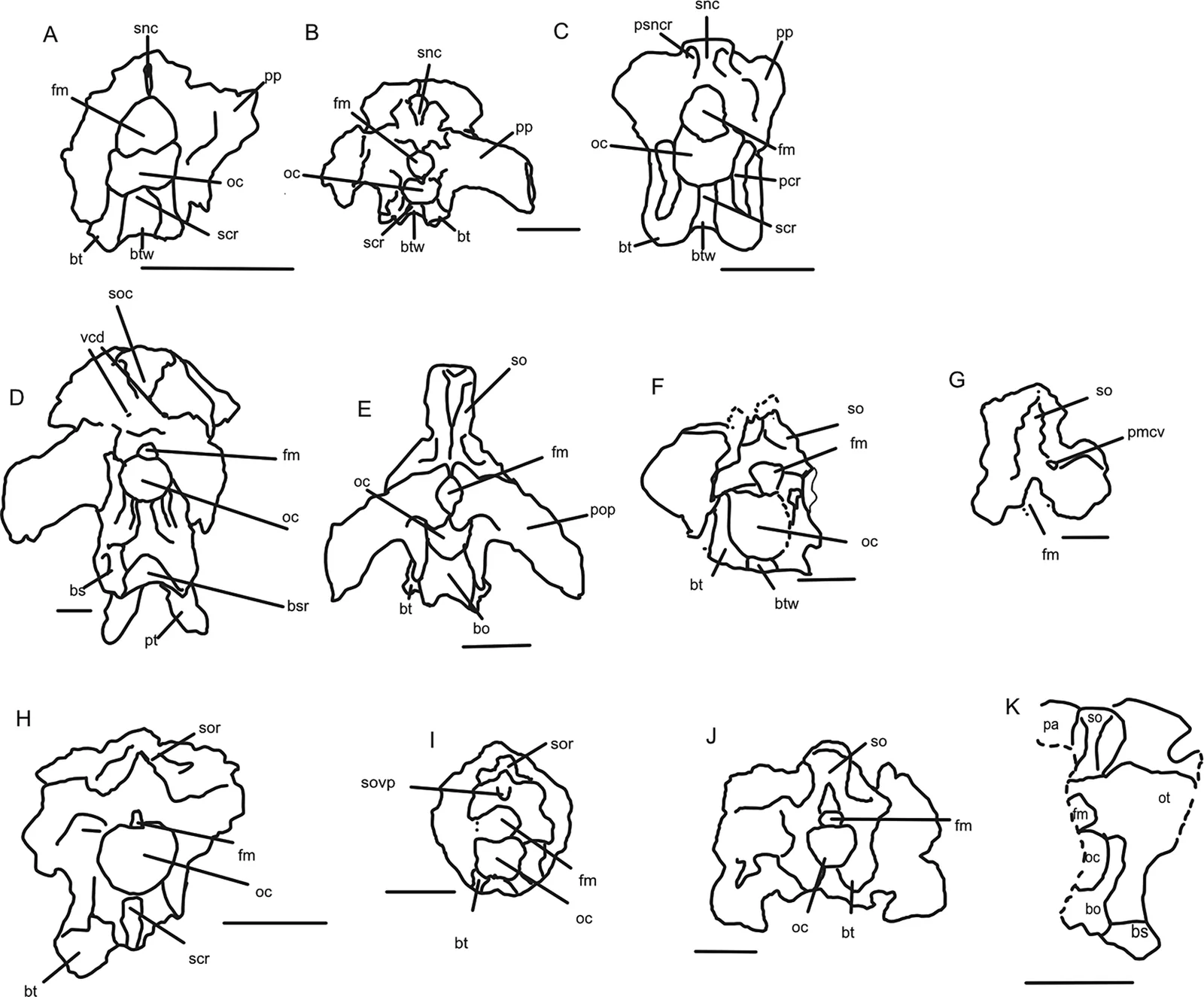
Comparisons of the posterior views of all known megaraptoran and other theropod braincases with overlapping elements with *Invictibellatrix* (NHMUK PV R6775). A: *Megaraptor* (adapted from [46]); B: *Murusraptor* (adapted from [31]); C: *I. amberina* gen. et sp. nov; D: *Acrocanthosaurus* (adapted from [47]); E: *Allosaurus* (adapted from [48]); F: *Carcharodontosaurus* (adapted from [49]); G: *Tameryraptor* (adapted from [50]); H: *Shaochilong* (adapted from [51]); I: *Timurlengia* (adapted from [52]); J: *Xiongguanlong* (adapted from [53]); K: *Guanlong* (adapted from [54]). Abbreviations: bo, basioccipital; bs, basisphenoid; bsr, basisphenoid recess; bt, basal tubera; btw, basituberal web; fm, foramen magnum; oc, occipital condyle; ocs, occipital condyle scar; ot: otoccipital; pmcv, posterior exit of middle cerebral vein; pa, parietal; pcr, paracondylar recess; pp, paraoccipital process; psncr, parasagittal nuchal crest recess; pt, pterygoid contact; scr, sub-condylar recess; snc, parasagittal nuchal crest; so, supraoccipital; sor, supraocciptal ridge; sovp, supraoccipital ventral process; vcd, vena capita dorsalis. Annotations are based on and limited to the original authors' descriptions of the specimens. Scale bars equal 50 mm.

**Etymology:** the generic name *Invictibellatrix* is derived from the Latin ‘*invictus*’ (‘unconquered’) and ‘*bellatrix*’ (‘warrior’ in the feminine form) and honours the future role of Megaraptora as Gondwanan apex predators, while early on in their evolutionary history they were smaller, non-apex predators [55–57]. This name is to give hope and strength to all those who are bullied, discriminated, victimized and oppressed that tomorrow will be theirs and not to give up in the face of such odds. The species epithet *amberina* is derived from the amber present in the plant debris beds of the Wessex Formation [58].

**Locality and horizon:** Sudmoor Point, near Brook, southwestern coast of the Isle of Wight, UK; Wessex Formation, Barremian, Lower Cretaceous. Most likely from the lower plant debris beds (L1 to L5).

**Holotype**: NHMUK PV R6775, a partial braincase.

**Diagnosis**: *Invictibellatrix amberina* can be diagnosed by the following unique character combination (autapomorphies marked with an asterisk): (i) *presence of a small basipterygoid recess anterior to the anterior tympanic recess (ATR) on each side of the parabasisphenoid, (ii) *slightly transversely compressed, hourglass-shaped nuchal crest, (iii) the presence of two large, deep and dorsoventrally elongate paracondylar recesses on the ventrolateral sides of the basioccipital, and (iv) *parasagittal nuchal crest recesses that are slightly curved and pronounced.

### 3.2. Anatomical description and comparisons

#### 3.2.1. Preservation and overall morphology

*Invictibellatrix amberina* is represented by a beach-rolled, rather poorly preserved and incomplete braincase (table 2) that shows no signs of plastic deformation. Everything anterior to the laterosphenoids has been lost; the basipterygoid, cultriform and paraoccipital processes are missing, and most of the skull roof is absent (figures 1–3). Although the braincase is extensively fused, the preserved elements include the basioccipital, exoccipitals, basisphenoid, parasphenoid, opisthotic, prootic and possible fragments of the laterosphenoid. Many of the neurocranial foramina and pneumatic fossae can be observed. While the specimen is beach-rolled and the surfaces are worn, based on the µCT scans, the lack of exposed internal bone structure on the surface and the preservation of neurocranial foramina, these have not affected the overall morphology, such as the autapomorphies or characters that have been scored, nor disproportionately affected any portion relative to others, i.e. weathering has not affected any of the key diagnostic features.

**Table 2.** Measurements of the holotype (NHMUK PV R6775) of *I. amberina* gen. et sp. nov. Measurements are in mm.

	transverse width	dorsoventral height	anteroposterior length
occipital condyle	37	26	-
foramen magnum	30	33	-
paracondylar recess	15	43	-
right vagal canal	5	5	-
nuchal crest	-	4	37

The endocast anatomy of *Invictibellatrix* (figure 4) is consistent with that of other theropods in that the posterior part of the endocast is sinusoidal, resembling the endocast morphology in theropod clades, such as Spinosauridae and Tyrannosauroidea, whereas in ornithischians (e.g. *Mantellisaurus*) the posterior portion is linear [59]. Additionally, the presence of tympanic and basisphenoid recesses indicates theropod affinities for the braincase [60]. Based on the overall proportions of the preserved part, the braincase of *Invictibellatrix* was originally slightly longer anteroposteriorly than dorsoventrally tall, as in *Allosaurus*, abelisaurids and tyrannosaurids [61–63]. This differs from the braincases of spinosaurids, which are dorsoventrally taller than they are anteroposteriorly long [12,25]. The sutures between the individual bones are not visible, even in the µCT scans, indicating that the individual is at least a sub-adult [64]. Measurements of the braincase are provided in table 2. This specimen is similar in size to the corresponding region in *Timurlengia euotica* [52], suggesting that the holotypic individual of *Invictibellatrix* was of a comparable body size to *T. euotica* (approx. 200–300 kg; ≤4 m length). As other megaraptorans are known to vary between 6–10+ m in length and likely over 2000 kg in body mass, this would make *Invictibellatrix* among the smallest, if not the smallest, megaraptoran discovered so far [39,65–67].

**Figure 4.**
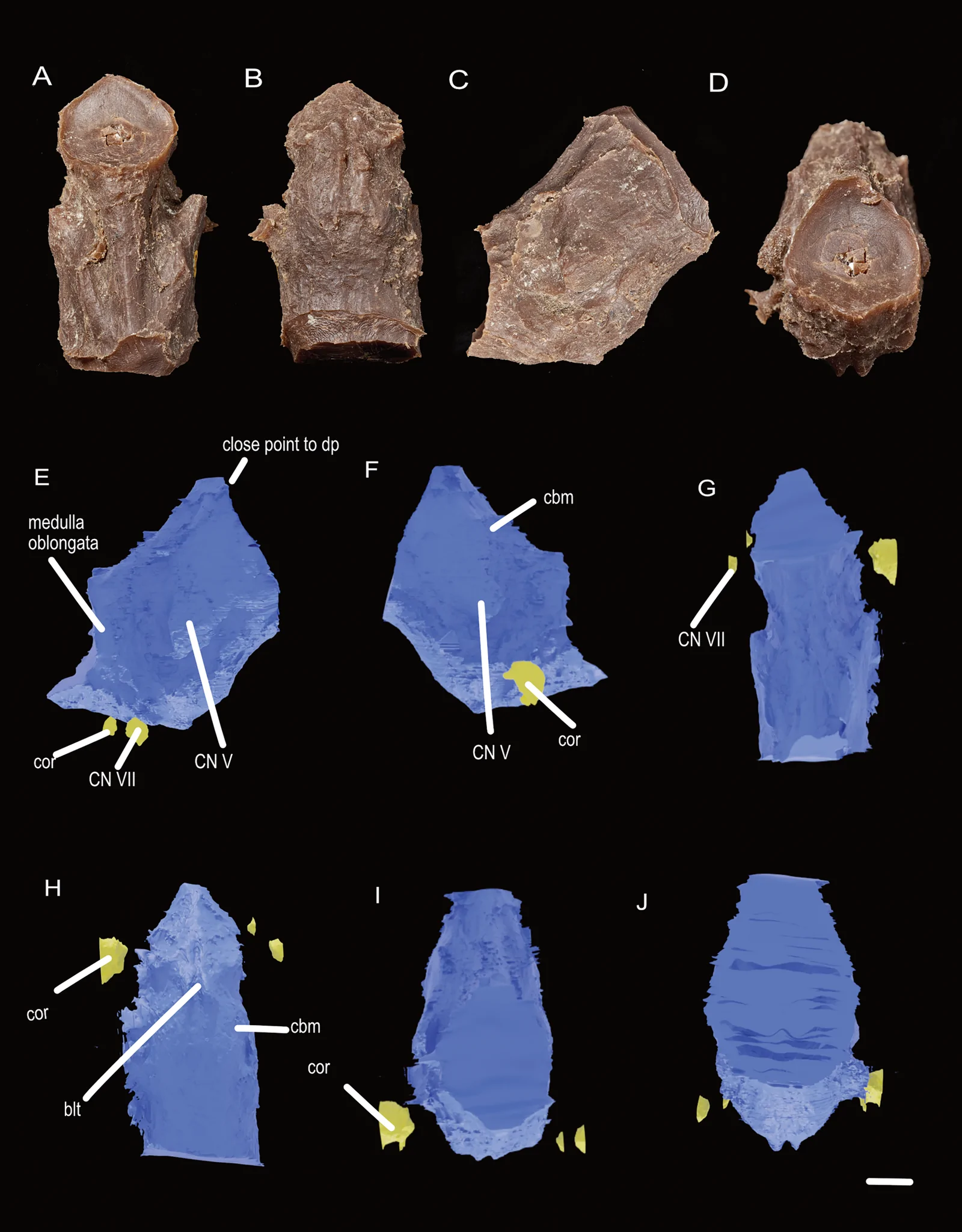
(A to D) Photographs of the physical endocast and (E to J) digital reconstruction of *I. amberina* gen. et sp. nov. in (A) dorsal, (B) ventral, (C) right lateral, (D) posterior, (E) right lateral, (F) left lateral, (G) dorsal, (H) ventral, (I) anterior and (J) posterior views. Abbreviations: blt, bilateral trough; cbm, cerebellum; CN V, VII, cranial nerves V, VII; cor, columnar recess; dp, dural peak. Scale bar equals 10 mm.

#### 3.2.2. Basioccipital, occipital condyle and foramen magnum

As the sutures between the individual bones have been obliterated, the contributions of the basioccipital and exoccipitals to the occipital condyle are unclear. While the specimen is weathered, this has not affected the overall morphology, e.g. the occipital condyle to foramen ratio, as shown by the internal structure revealed by the µCT scans and examination of the specimen. The occipital condyle is transversely broader than dorsoventrally high and has a rounded sub-rectangular shape in posterior view. Its articular surface is separated from the main body of the basioccipital by a short, stout and slightly waisted neck. As in all theropods other than derived maniraptorans [60], the occipital condyle is slightly wider transversely than the foramen magnum. The foramen magnum is almost oval in outline, narrowing dorsally, with the long axis of the oval oriented dorsoventrally. Its ventral rim invaginates the dorsal margin of the occipital condyle. The morphology of this region in *Invictibellatrix* closely resembles that of *Murusraptor* (figure 3; [31]). *Invictibellatrix* does not preserve clearly overlapping braincase elements with the megaraptorid *Joaquinrapto*r [65], precluding comparisons.

This combination of characters differs from that present in early-diverging theropods, in which the occipital condyle is dorsoventrally compressed and reniform, as in *Zupaysaurus, Megapnosaurus, Sinosaurus, Dilophosaurus* and *Cryolophosaurus*, and from the condition present in the early-diverging tetanuran *Piatnitzkysaurus*, which has a rhomboidal foramen magnum [60,61,68–71]. In abelisaurids, there is some variation in occipital condyle shape, being sub-spherical in many taxa (*Llukalkan, Majungasaurus, Carnotaurus, Abelisaurus*, *Viavenator* and *Aucasaurus*; [72,73]). Within Spinosauridae, the occipital condyle is almost spherical and there is a semicircular foramen magnum [12,19]. Carcharodontosaurids differ from *Invictibellatrix*, as *Acrocanthosaurus* has an oval foramen magnum with a transverse long axis and *Shaochilong* has a sub-rectangular dorsoventrally oriented foramen magnum, whereas that of *Tameryraptor* is incompletely preserved and *Carcharodontosaurus iguidensis* has a sub-trapezoidal foramen magnum that may have been affected by taphonomy to a greater extent than the other specimens (figure 3; [49]). The occipital condyles of *Acrocanthosaurus* and *Shaochilong* have a sub-circular occipital condyle in posterior view, whereas that of *C. iguidensis* is sub-square in posterior view and that of *Tameryraptor* is unknown (figure 3; [49,74–76]).

The proceratosaurid tyrannosauroid *Guanlong* has a circular occipital condyle with a circular to rhomboidal foramen magnum (figure 3; [54,77]). Late-diverging non-tyrannosaurid tyrannosauroids (e.g. *Timurlengia* and *Xiongguanlong*), non-tyrannosaurine tyrannosaurids (e.g. *Alioramus*) and tyrannosaurines (e.g. *Daspletosaurus*) have rounded rectangular occipital condyles that are transversely wider than dorsoventrally tall, along with an elliptical foramen magnum that is transversely wider than dorsoventrally tall (figure 3; [52–54,61]). In *Struthiomimus* (NHMUK PV R6784 (cast)) and an indeterminate ornithomimid [78], the occipital condyle is a rounded rectangle that is transversely wider than it is dorsoventrally tall, with an almost semicircular foramen magnum. In *Nothronychus*, the occipital condyle is a long ellipse that is transversely wider than dorsoventrally tall, with a circular foramen magnum [79]. In later-diverging coelurosaurs, such as *Troodon*, the occipital condyle is almost square, but slightly wider than tall [80], with a circular foramen magnum, as is also seen in the alvarezsauroid *Mononyku*s, other troodontids, such as *Sinovenator*, and some dromaeosaurids, such as *Tsaagan* [81–84]. *Invictibellatrix* has a foramen magnum transverse width to occipital condyle transverse width ratio (FOR) of 0.8. Abelisaurids have a FOR range of 0.56–0.82, spinosaurids 0.65–0.83, carcharodontosaurids 0.36–0.53, tyrannosaurids 0.27–0.59 and megaraptorans 0.68–0.88: maniraptoriforms have a FOR greater than 1.0, as do some early-diverging theropods (table 3).

**Table 3.** Foramen magnum transverse width to occipital condyle transverse width ratios (FOR) for selected theropod dinosaurs.

clade	clade	taxon	FOR	source
Allosauroidea	Allosauridae	*Allosaurus fragilis*	0.60	[62]
Allosauroidea	Allosauridae	*Allosaurus jimmadseni*	0.61	[48]
Allosauroidea	Carcharodontosauridae	*Acrocanthosaurus*	0.36	[47]
Allosauroidea	Carcharodontosauridae	*Carcharodontosaurus*	0.53	[49]
Allosauroidea	Carcharodontosauridae	*Giganotosaurus*	0.40	[74]
Allosauroidea	Carcharodontosauridae	*Shaochilong*	0.28	[51]
Allosauroidea	Metriacanthosauridae	*Sinraptor*	0.43	[85]
Ceratosauria	Abelisauridae	*Arcovenator*	0.47	[86]
Ceratosauria	Abelisauridae	*Carnotaurus*	0.56	[87]
Ceratosauria	Abelisauridae	*Guemesia*	0.80	[88]
Ceratosauria	Abelisauridae	*Llukalkan*	0.75	[73]
Ceratosauria	Abelisauridae	*Majungasaurus*	0.65	[63]
Ceratosauria	Abelisauridae	*Viavenator*	0.82	[72]
Ceratosauria	Abelisauroidea	*Eoabelisaurus*	0.50	[89]
Ceratosauria	Noasauridae	*Limusaurus*	1.02	Stiegler, 2023 (images per comms)
Ceratosauria	Noasauridae	*Masiakasaurus*	1.00	[90]
Ceratosauria	Ceratosauridae	*Ceratosaurus*	0.45	[91]
Maniraptoriformes	Alvarezsauroidea	*Haplocheirus*	1.12	[92]
Maniraptoriformes	Ornithomimosauria	Ornithomimidae indet.	1.01	[78]
Maniraptoriformes	Therizinosauria	*Falcarius*	1.13	[93]
Megalosauroidea	Megalosauridae	*Dubreuillosaurus*	0.77	[94]
Megalosauroidea	Megalosauridae	*Eustreptospondylus*	0.74	Measured from unpublished CT data
Megalosauroidea	Megalosauridae	*Piatnitzkysaurus*	0.70	[60]
Megalosauroidea	Spinosauridae	*Baryonyx*	0.83	[25]
Megalosauroidea	Spinosauridae	*Ceratosuchops*	0.65	[12]
Megalosauroidea	Spinosauridae	*Irritator*	0.82	[95]
Megalosauroidea	Spinosauridae	*Riparovenator*	0.67	[12]
Megalosauroidea	Spinosauridae	*Suchomimus*	0.70	Hendrickx, 2023 (images per comms)
Neotheropoda		*Cryolophosaurus*	0.44	[68]
Neotheropoda		*Dilophosaurus*	0.87	Marsh, 2023 (images per comms)
Neotheropoda		*Sinosaurus*	0.59	[71]
Neotheropoda		*Zupaysaurus*	1.11	[61]
Tyrannosauroidea	Megaraptora	*Megarapto*r	0.88	[46]
Tyrannosauroidea	Megaraptora	*Murusraptor*	0.68	[31]
Tyrannosauroidea	Megaraptora	*Invictibellatrix*	0.80	NHMUK PV 6775, this study
Tyrannosauroidea	Proceratosauridae	*Guanlong*	0.62	[54]
Tyrannosauroidea	Tyrannosauridae	*Alioramus*	0.59	[54]
Tyrannosauroidea	Tyrannosauridae	*Bistahieversor*	0.40	[96]
Tyrannosauroidea	Tyrannosauridae	*Daspletosaurus*	0.40	[61]
Tyrannosauroidea	Tyrannosauridae	*Gorgosaurus*	0.45	TMP 1994.143.1
Tyrannosauroidea	Tyrannosauridae	*Teratophoneus*	0.37	[97]
Tyrannosauroidea	Tyrannosauridae	*Tyrannosaurus*	0.27	[98]
Tyrannosauroidea		*Timurlengia*	0.99	[52]
Tyrannosauroidea		*Xiongguanlong*	0.43	[53]

Ventral to the occipital condyle, the posterior surface of the basioccipital possesses a relatively deep sub-condylar recess that extends from the ventral margin of the condylar neck, expanding slightly transversely as it descends, separating the basal tubera. The base of this recess is confluent with a midline notch on the ventral margin of the basioccipital (= basioccipital crest) that further separates the basal tubera. The basal tubera are connected by a ‘web’, similar to the condition in *Piatnitzkysaurus* [60], and that is likely homologous to the ‘basituberal web’ described in *Baryonyx, Irritator* and *Murusraptor* [25,31,95], and the ‘intertuberal lamina’ described in *Megaraptor* [46]. The posterior surface of the basituberal web in *Invictibellatrix* is concave in all directions, especially transversely. This resembles *Megaraptor* [46] but differs from *Murusraptor* in which the basituberal web is posteriorly convex (figure 3; [31]). *Invictibellatrix* shares with *Megaraptor* and *Murusraptor* the condition of triangular basituberal webbing dorsoventrally expanding from the base of the occipital condyle (figure 3; [31,46]). The sub-condylar recess of *Invictibellatrix* is defined by well-developed ridges that extend from the lateral margins of the occipital condyle and merge with the basal tubera, similar to the condition seen in *Murusraptor* and *Megaraptor* [31,46]. The basituberal web is similar in shape in *Allosaurus* [48] but starts from the lower lateral margin of the occipital condyle, rather than directly below the occipital condyle neck as in megaraptorans. The basituberal web and sub-condylar recesses are absent in *Carnotaurus* [99] and *Nothronychus* [79], with only an incipient basituberal web present in *Sinovenator* [100]. In the spinosaurid *Irritator*, the webbing is elliptical, whereas in *Riparovenator* and *Ceratosuchops* it is triangular, but in all spinosaurids the sub-condylar recess is transversely divided, and the basituberal web starts between one-third and halfway down the recess; by contrast, in megaraptorans it starts from directly below or lateral to the occipital condyle. In *Acrocanthosaurus*, the basituberal web starts at the lower lateral margins of the occipital condyle but is more trapezoidal in shape than triangular (figure 3; [47]). *Daspletosaurus* has triangular webbing but the most dorsal margin of the sub-condylar recess starts at the very base of the occipital condyle neck, differing from megaraptorans wherein the most dorsal margins of the sub-condylar recesses are significantly higher [61].

In *Invictibellatrix*, the basituberal web connects the preserved parts of the basal tubera for most of their length except for their distal-most ends, which are free and form blunt, semicircular processes in posterior view. The ridges forming the lateral margins of the sub-condylar recess separate the basituberal web from two large, deep and dorsoventrally elongate paracondylar recesses on the ventrolateral sides of the basioccipital. These paracondylar recesses retain similar widths and depths along their entire lengths. They have narrow reniform outlines, with the ventral part being slightly curved laterally. Paracondylar recesses are well-developed in *Megaraptor* and are only weakly developed in *Murusraptor* (figure 3; [31,46,101]). Among other theropods, these recesses are absent in *Carnotaurus* [99] and *Irritator* [102], whereas they are weakly developed and do not extend as far ventrally as the base of the basal tubera in *Ceratosuchops* and *Riparovenator* [12]. These recesses are also absent in the carcharodontosaurid *Acrocanthosaurus*, the tyrannosaurid *Daspletosaurus* [61,76], the non-tyrannosaurid tyrannosauroids *Stokesosaurus* and *Xiongguanlong* ([53,103]), and the therizinosaurid *Nothronychus* [79], in addition to dromaeosaurids and various other derived coelurosaurs [52]. The deep paracondylar pockets are likely comparable with paracondylar recesses that are present in the tyrannosaurids *Alioramus*, *Tyrannosaurus* and * Teratophoneus* [54]. The paracondylar recesses in *Invictibellatrix* are framed by a sharp ridge on the otoccipital dorsally and by the prominent crista tuberalis laterally.

In lateral view, the basioccipital forms the posterior part of the ventral floor of the endocranial cavity. The precise boundary between the basioccipital and the otoccipital is unclear owing to fusion but can be approximated from the positions of the cranial nerve foramina and the inner ear. The margins of the basioccipital are strongly ridged to form the articular surface for the otoccipital complex. In ventral view, the outline of the basioccipital is sub-triangular. Anteriorly, the basal tubera are connected by the basituberal web terminating ventrally in a moderately thick basioccipital crest that forms the posterior margin of the basisphenoid recess.

#### 3.2.3. Supraoccipital

In lateral view, the supraoccipital is anterodorsally inclined with respect to the rest of the posterior skull surface, similar to *Murusraptor* [31], but differing from *Megaraptor* in which it is posterodorsally inclined [46]. The absence of visible sutures makes it difficult to determine the boundary between the parietals, supraoccipital and otoccipitals. NHMUK PV R6775 preserves the lower part of an eroded supraoccipital knob, with the widest point being 30 mm in breadth, with a similar feature present in *Murusraptor* [31] and *Megaraptor* (figure 3; [101]). A supraoccipital knob appears to be present in the tyrannosauroids *Xiongguanlong* and *Timurlengia*, but absent in *Guanlong* and *Dilong* (figure 3; [52]). Additionally, it is present in *Giganotosaurus* [74] and *Acrocanthosaurus* [47], where it is more rectangular in the latter, but it is absent in *Allosaurus* [48], *Carnotaurus* [99], *Irritator, Ceratosuchops, Daspletosaurus* [61] and *Nothronychus* [79].

An abraded nuchal crest is present (figure 1), similar to the crests in *Murusraptor* and *Megaraptor* (figure 3; [31,46]). The shallow recesses on either side of the parasagittal nuchal crest give it a slightly compressed hourglass-shaped outline, which differs from the teardrop-shaped nuchal crest in *Murusraptor*, which lacks the shallow recesses [31] and the rectangular shape present in *Megaraptor* [46]. Nuchal crests are present in the late-diverging tyrannosauroids *Xiongguanlong* and *Timurlengia,* are absent in *Guanlong* and *Dilong* (figure 3; [52]), and form a double ‘boss’ in the carcharodontosaurids *Giganotosaurus* and *Acrocanthosaurus* (figure 3; [47]). It is unclear how much the supraoccipital contributes to the dorsal margin of the foramen magnum in NHMUK PV R6775 because of its fusion with the otoccipitals. As the nuchal crest is abraded and the supraoccipital is incomplete, the presence/absence of a supraoccipital midline ridge as in *Xiongguanlong* [52] is unknown.

#### 3.2.4. Otoccipital

As with most post-hatchling archosaurs [63], the exoccipital is fused indistinguishably with the opisthotic, forming the otoccipital complex and the junction between them cannot be observed, even in µCT data. In posterior view, the otoccipital forms the lateral (and probably dorsal) margin of the foramen magnum and connects to what remains of the prootic and paraoccipital processes. This bone complex contacts the supraoccipital dorsomedially, the basioccipital ventromedially, the prootic anteriorly and the parietal dorsally. Only the bases of the paraoccipital processes are present, which are preserved as rounded expansions. The larger right base has a square outline, whereas the left base is more spherical, yet this is most likely an artefact of preservation such that either side may not be indicative of the original shape of the paraoccipital processes. On both sides of the braincase, a single opening is present in the dorsal-most part of the paracondylar recess, situated on the ventrolateral side of the neck of the occipital condyle, in the posteroventral-most part of the otoccipital, which is likely the external opening for the vagal canal (for CN X-XI). This resembles the condition in *Murusraptor*, in which CN IX separates from CN X-XI before exiting the braincase [31]. The vagal canals are partially infilled with matrix, although the right canal was prepared to a greater extent and has a diameter of 5 mm. From the dorsolateral contribution of the otoccipital to the occipital condyle, a small overhanging ridge runs transversely across each paracondylar recess, forming its dorsal margin. A similar ridge or change in slope appears to be present in both *Megaraptor* and *Murusraptor* [31,46], but is absent in *Carnotaurus* [99], *Daspletosaurus* [61], *Acrocanthosaurus* [47], *Stokesosaurus* [103] and *Nothronychus* [79]. A comparable ridge is present in *Ceratosuchops, Ripravenator* and *Irritator*, but opens more ventrally in relation to the occipital condyle in those taxa. The courses of the vagal canals cannot be traced internally, even in the µCT scans, and they are lost a short distance within the bone. There is no sign of an opening for CN XII on the posteroventral part of the otoccipital, which may be owing to damage or poor preservation. Small indentations on the lateral surfaces of the basioccipital condyle neck are present but unlikely to represent these openings; they are too indistinct to be certain. The fenestra ovalis is present, being circular in lateral view and situated posterior to the foramen for CN VII. However, preparation or poor preservation appears to have obliterated any sub-dividing sutures/struts in the fenestra ovalis, such as the crista interfenestralis, which are not visible in the µCT scans.

In lateral view, the anterior portion of the otoccipital is dorsoventrally expanded, tapering posteriorly to form the base of the paraoccipital process. The columellar recess is almost circular and is posteroventral to CN VII. Parts of the inner ear are present, but they are poorly preserved, precluding accurate segmentation and reconstruction. Although present, the partially preserved semicircular canals are too fragmentary to be included in the endocast reconstruction (see below). A stout metotic strut connects the base of the paraoccipital processes to the posterior part of the otoccipital complex (crista tuberalis), separating the lateral and posterior surfaces of the braincase as in most theropods, except carcharodontosaurids [74].

#### 3.2.5. Parabasisphenoid

The basisphenoid and parasphenoid are indistinguishably fused, with no visible sutures even in the µCT scans, so we refer to this combined structure as the parabasisphenoid. Similar to other theropods [63], the parabasisphenoid complex contacts the otoccipital posterodorsally, the prootic dorsally and the basioccipital posteriorly. The parabasisphenoid forms the anterior and ventral part of the braincase, along with the basal tubera. Although the boundary between the parabasisphenoid and basioccipital is not visible, it is usually at the level of the fenestra ovalis, near the columellar recess. The parabasisphenoid is widest posteriorly at the level of the basal tubera and gradually narrows anteriorly towards the base of the cultriform process, where there is a slight expansion again. The basisphenoid recess is deep, has a circular outline in ventral view and opens directly ventrally. This is similar to *Murusraptor*, wherein the basisphenoid recess also opens ventrally and is deeply evacuated [31], but distinguishes it from *Megaraptor* in which it has a more funnel-like shape [46], with the latter morphology also characterizing tyrannosauroids, such as *Bistahieversor* [104], and carcharodontosaurids, such as *Acrocanthosaurus* and *Carcharodontosaurus* [47].

Two shallow pneumatic recesses are present on the lateral surfaces of the parabasisphenoid. The depth and extent of these recesses are similar on the right and left sides, but they are better preserved on the right. The posterior recess, the ATR, is the largest. The ATR is sub-elliptical in outline, with the long axis of this ellipse extending dorsoventrally, and it extends posteroventrally from a point approximately 20 mm ventral to the foramen for CN V. It is comparable in shape with that of *Murusraptor* [31]. It is possible that the ATR extended dorsally to the ventral part of the prootic, but this cannot be confirmed as the sutures are not visible. Anterodorsally, the ATR is overhung laterally by a tab-shaped process, which likely represents the ala basisphenoidalis (= preotic pendant) of *Invictibellatrix*. Several deep sub-recesses are visible within the ATR. In the posterodorsal corner of *Invictibellatrix*, there is a sub-circular sub-recess (11 mm wide and 7 mm tall on the right side, 6 mm in diameter on the left side: this discrepancy may be a result of preservation and preparation). Ventrally on the right side, a partially infilled circular sub-recess with a diameter of 6 mm opens into the ATR. A small elliptical foramen or recess (3 mm wide and 6 mm tall), with its long axis oriented almost vertically, is situated in the posteroventral base of the ala basisphenoidalis/preotic pendant on the right side, separated from the ATR by a thin wall of bone. On the left-hand side, a circular foramen with a diameter of 6 mm is in a similar position, separated by 7 mm from the basisphenoid recess, which may be owing to crushing.

A smaller recess (basipterygoid recess?) is present anterior to the parabasisphenoid recess on each side of the parabasisphenoid, situated posterodorsal to the broken base of the cultriform process. These basipterygoid recesses are elliptical in shape and extend posteroventrally, gradually shallowing in that direction, with heights of approximately 22 mm and widths of approximately 8 mm at their greatest extents. Each recess is separated from the ATR by a stout, posteroventrally oriented ridge, the dorsal termination of which lies adjacent to the ventral-most projection of the ala basisphenoidalis. This recess is not present in *Murusraptor* [31], and the relevant area is not preserved in *Megaraptor* [46].

In anterior view, the base of the cultriform process has an equilateral triangular shape, like that of *Murusraptor* [31]. The relevant region is not preserved in *Megaraptor* [46]. Approximately 10 mm above the dorsal margin of the broken cultriform process are the ventral rim of the pituitary fossa and the sella turcica, which are both exposed owing to breakage. The foramina that normally pierce the sella turcica (for CN VI and the anterior carotid arteries) are not visible. Lateral to the pituitary fossa are two circular foramina, each around 6 mm in diameter, which most likely represent the exits for the sphenoidal arteries.

#### 3.2.6. Prootic and laterosphenoid

Most of the prootic is preserved, forming part of the dorsolateral wall of the braincase, along with the partially preserved laterosphenoid. There are no visible sutures, but the prootic likely contacted the parabasisphenoid ventrally, the laterosphenoid anterodorsally and the otoccipitals posteriorly. Its anterior border and dorsal posterior process overhang the CN V foramen anteriorly and dorsally. On the lateral side of the braincase, the basisphenoid-prootic suture is not visible, but the presence of the preotic pendant indicates the limits of the bones, as in most other theropods [31]. The prootic-laterosphenoid suture is not visible, which is not uncommon in adult theropods [105]. Both posterior foramina openings of CN V are taller than wide, with the right one being 12 mm by 9 mm and the left one 14 mm by 12 mm. The former is elliptical in shape with infilled sediment, and the latter is more spherical which could be a result of erosion and/ or preparation and passes into the endocranial cavity. The foramen for cranial nerve VII on the right lateral posterior opening foramen is 7 mm wide and 2.5 mm tall, forming a long ellipse. Owing to erosion on the left side, the foramen for CN V is damaged internally, preventing any accurate reconstruction of its shape. None of the cranial nerves’ routes can be reconstructed continuously through the braincase to the endocast, but there are openings for CN V (on the right lateral side) and CN VII (on the left lateral side) that have had limited deformation or expansion. However, on the left lateral wall of the braincase, the cranial nerve margins have been broken by either erosion or preparation. This has increased and damaged the internal opening of CN V, affecting its preserved structure. The lateral surface of the prootic is orientated ventrolaterally and is divided into two regions by the foramen for CN V, with the anterior region being elliptical in outline with its dorsal rim terminating in the preotic pendant. This rim overhangs both the anterior part of the ATR and the basisphenoid recess, terminating dorsally level with the foramen for CN VII.

### 3.3. Key anatomical features nomenclature

For the sake of clarity, the definitions used in the following descriptions are used to describe key features between potentially closely related taxa (figure 3). The paracondylar recesses are those that are parallel or sub-parallel to the basal tubera and can continue to the occipital condyle. The sub-condylar recess is formed between the basal tubera and the occipital condyle and is a deep recess in the ventral surface of the braincase and not just formed by the basituberal web as in early tyrannosauroids [52]. The parasagittal nuchal crest is a crest/ knob on the supraoccipital in posterior view (variously referred to as the nuchal process [74] or supraoccipital knob [63]). The parasagittal nuchal crest of the supraoccipital can terminate in a supraoccipital ridge, i.e. forming a distinct linear feature. The parasagittal nuchal crest recesses are the recesses on either side of the parasagittal nuchal crest of the supraoccipital crest between the nuchal crest and the supraoccipital.

### 3.4. Detailed anatomical comparisons with key taxa

The parasagittal nuchal crest of the supraoccipital (also known as the pronounced nuchal process [74] or supraoccipital knob [63]) is present but abraded in *Invictibellatrix*, *Murusraptor* and *Megaraptor*, with *Invictibellatrix* having a slightly compressed hourglass-shaped outline, whereas *Murusraptor* has a tear-shaped one and it is rectangular in *Megaraptor* [31,46]. In *Shaochilong,* the nuchal crest is especially prominent, three times the width of the foramen magnum in addition to thickening transversely and anteroposteriorly when viewed dorsally, like *Acrocanthosaurus* and *Carcharodontosaurus* [51], where it also forms a double ‘boss’ in *Acrocanthosaurus* [47]. The parasagittal nuchal crest in *A. jimmadseni* and *fragilis* becomes lower and narrower ventrally [48], differing from the Megaraptora. The nuchal crest is absent *in Guanlong*; a slight nuchal crest that is wider than the foramen magnum is present in *Xiongguanlong*, and similar to the diamond-shaped one in *Timurlengia* [52]. The nuchal crest terminates in a supraoccipital ridge in *Shaochilong*, *Timurlengia* and *Xiongguanlong,* but this is not preserved or present in the Megaraptoran specimens (figure 3). Yet the lack of preservation or genuine absence of the ridge does not affect the comparisons or shape of the nuchal crests.

Parasagittal nuchal crest recesses are present on either side of the sagittal nuchal crest and are slightly curved in *Invictibellatrix*, but these are absent in both *Megaraptor* and *Murusraptor*. Comparable recesses are not present in *Acrocanthosaurus*, *C. iguidensis* and *Shaochilong* with slight recesses/depressions present in *A. jimmadseni* that are more rectangular than in *Invictibellatrix* and potential recesses in *Tameryraptor* but these are triangular (figure 3)*.* In the early-diverging tyrannosauroids, there are very shallow recesses in *Xiongguanlong* that are linear to curved in shape, like *Timurlengia,* but absent in *Guanlong.* The shape, symmetry and depth of these recesses in *Invictibellatrix* suggest that these are not just an artefact of taphonomy but a genuine feature.

In *Invictibellatrix,* the paracondylar recesses are large, dorsoventrally elongated with reniform outlines and the ventral parts are slightly curved laterally. The recesses are well-developed in *Megaraptor* but not as ventrally extended; this might be owing to the lack of preservation of the basioccipital and are only weakly developed in *Murusraptor* (figure 3)*.* Within the carcharodontosaurids, the paracondylar recesses are unknown in *Tameryraptor* owing to the lack of preservation and in *C. iguidensis* the specimen is fragmentary and incomplete, so the presence of such recesses is inconclusive. The paracondylar recesses are present in *Acrocanthosaurus* but are not as distinct or defined as in *Invictibellatrix,* whereas in *Shaochilong* the ventral half is not as defined/deep, straighter and is angled more obtusely dorsoventrally in relation to the occipital condyle, differing from *Invictibellatrix*. *Allosaurus jimmadseni* has straighter and less prominent paracondylar recesses compared with *Invictibellatrix.* In the early-diverging tyrannosauroids: *Guanlong*, *Timurlengia* and *Xiongguanlong,* the paracondylar recesses are absent (figure 3).

*Invictibellatrix* shares a triangular-shaped sub-condylar recess that is narrower than the occipital condyle with *Megaraptor* and *Murusraptor* in which the basituberal webbing dorsoventrally expands from the base of the occipital condyle [31,46]. The presence of a sub-condylar recess is unknown in *Tameryraptor* as the portion of the braincase is not preserved, and in *C. iguidensis* as it is only partially preserved, and so the extent of the recess is not known (figure 3). *Acrocanthosaurus* has an extensive sub-condylar recess that is wider than the occipital condyle, incised and shaped as an inverted T or Y-shaped. In *Shaochilong,* the sub-condylar recess is only partially preserved, not as extensive as *Acrocanthosaurus* and appears to be narrower than the occipital condyle owing to only being partially preserved. Yet the sub-condylar recess in *Shaochilong* appears more similar to *Acrocanthosaurus* than the megaraptorans (figure 3). *Allosaurus jimmadseni* has a sub-condylar recess that is moderate but narrower than the occipital condyle, which is trapezoidal to triangular, being dorsoventrally taller than transversely wide. In early tyrannosauroids, the basituberal web is very short and flat, and the basioccipital is not invaded by a sub-condylar recess, hence is absent in *Guanlong*, *Timurlengia* and *Xiongguanlong,* but present in derived tyrannosaurids [52]. The short basituberal web is a feature of early-diverging tyrannosauroids, differing from *Invictibellatrix* and other megaraptorans.

Basipterygoid recesses are present in tyrannosauroids but change through ontogeny of the late-diverging taxa, such as *Daspletosaurus* and *Bistahieversor*, from a rectangular opening in juveniles to a slit-like window in adults, describing this as occurring within tyrannosauroids [54]. In *Guanlong*, *Timurlengia* and *Xiongguanlong,* the region is either not preserved and/or obscured. The section of braincase is not preserved in *Megaraptor*, whereas in *Murusraptor* the recess is a large oval foramen externally, which leads into an extensive pneumatic recess within the basisphenoid [31]. In *Invictibellatrix*, the recess is also oval shaped but is relatively shallow and does not appear to invade the basisphenoid, at least not to the same extent as in *Murusraptor*. The development and shape of this recess in *Invictibellatrix* differentiates it from both *Murusraptor* and tyrannosauroids, and we consider it a local autapomorphy of the new taxon. The relevant portion of the braincase where the basipterygoid recess would be present is not preserved in *Tameryraptor, C. iguidensis* and *Shaochilong,* so its presence in these taxa is unknown. In *Acrocanthosaurus,* the portion of the braincase is preserved but a basipterygoid recess is not present or not preserved [47], whereas in *A. jimmadseni,* the basipterygoid recess is large and ventrally unrestricted, differing from *Invictibellatrix.*

### 3.5. Endocast

A physical endocast was made soon after the acquisition of the specimen in the 1920s (figure 4), which is similar in appearance to the digital endocast generated from CT scans. The cranial endocast is partial, as the forebrain is missing; it starts anteriorly from posterior to the unpreserved dural peak (the point of greatest dorsal expansion of the endocast, where the dural sinuses and meninges are housed in life) and ends posteriorly at an incomplete medulla oblongata just above the occipital condyle (figure 4). The hindbrain is sub-horizontal, with a midbrain to hindbrain angle of around 55°, which is comparable with the angles observed in other non-maniraptoran theropods [31]. As in other non-maniraptoran theropods, there are no vascular impressions on the cranial endocast, indicating that the dorsal longitudinal venous sinus and dura mater were large enough to obscure the morphology of the encephalic structures, but the taphonomic conditions may not have been sufficient to preserve them [76,106,107]). Comparable with *Murusraptor* [31], the opening of CN V appears to be very closely aligned vertically or close to where the dural peak (dorsal expansion) would be, but it is slightly more posteriorly positioned in *Invictibellatrix*, unlike in carcharodontosaurids and ceratosaurians where the dural peak is more anterior relative to the CN V opening [63,91,108–110]. The extent of the midbrain is uncertain as it is incomplete. Although incomplete, some features of the hindbrain are visible, including the cerebellum, the medulla oblongata and a few foramina for cranial nerves. The cerebellum is not clearly expanded in the endocast. The medulla oblongata is incomplete, but its ventral surface has a bilateral trough that extends transversely from the sagittal plane. The base of CN V is visible but is not much more than a protrusion. The external openings for the right CN VII and both columellar recesses lead to circular canals that continue internally but could only be followed slightly internally.

**Figure 5.**
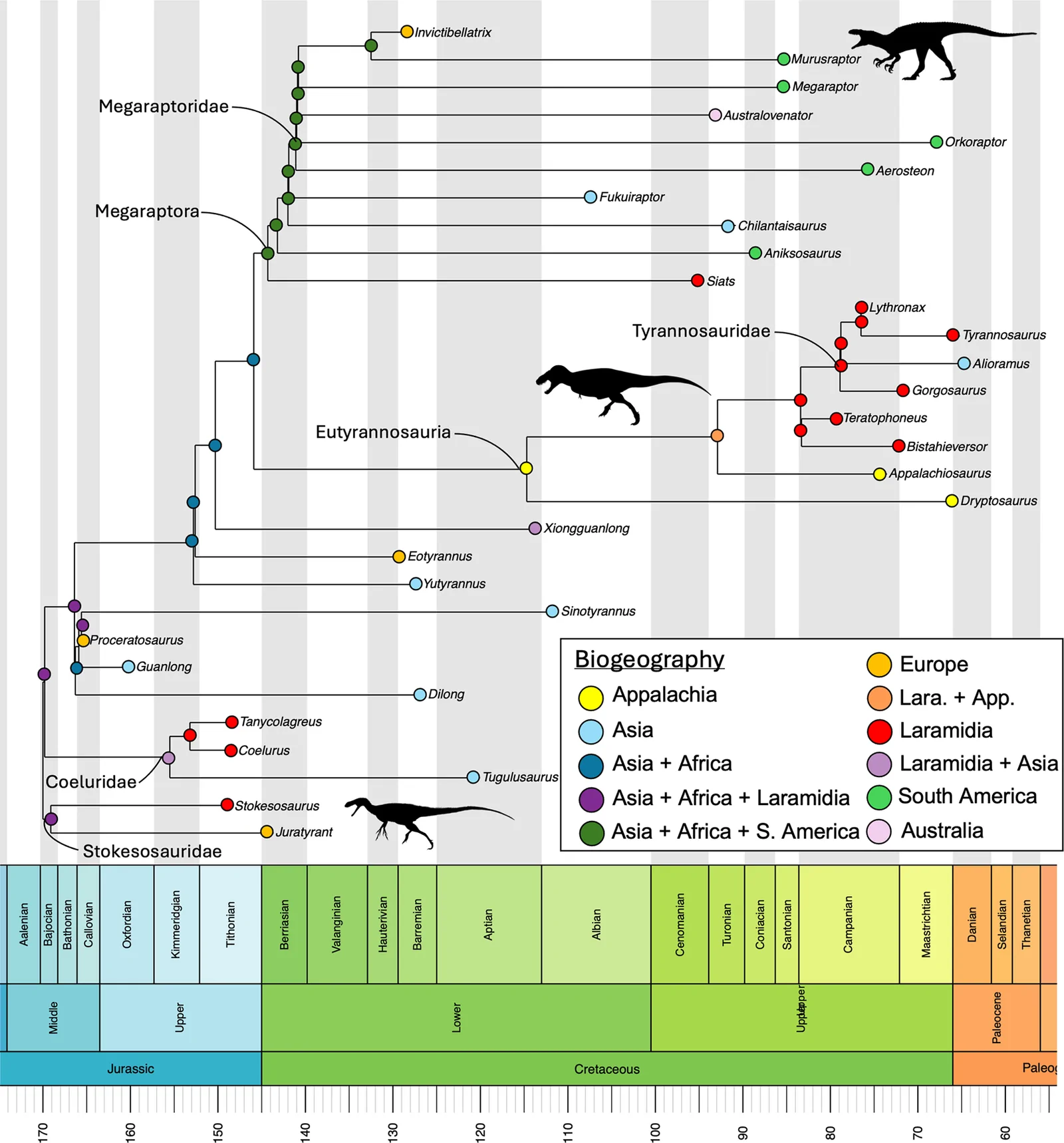
Time-calibrated strict consensus tree based on the Naish & Cau [24] data matrix under equal weights and biogeographical results of Tyrannosauroidea, including Megaraptora and *I. amberina*, including likely geographical origins. Lara. + app. refers to Laramidia and Appalachia. Silhouettes from Phylopic: *Australovenator wintonensis* by T. Tischler used under the License Attribution-ShareAlike 3.0 unported https://creativecommons.org/licenses/by-sa/3.0/*Tyrannosaurus rex* by Scott Hartman used under the Attribution-NonCommercial-ShareAlike 3.0 unported https://creativecommons.org/licenses/by-nc-sa/3.0/ and *Stokesosaurus clevelandi* by Scott Hartman (modified by T. Michael Keesey) used under the Attribution-NonCommercial-ShareAlike 3.0 unported https://creativecommons.org/licenses/by-nc-sa/3.0/.

**Figure 6.**
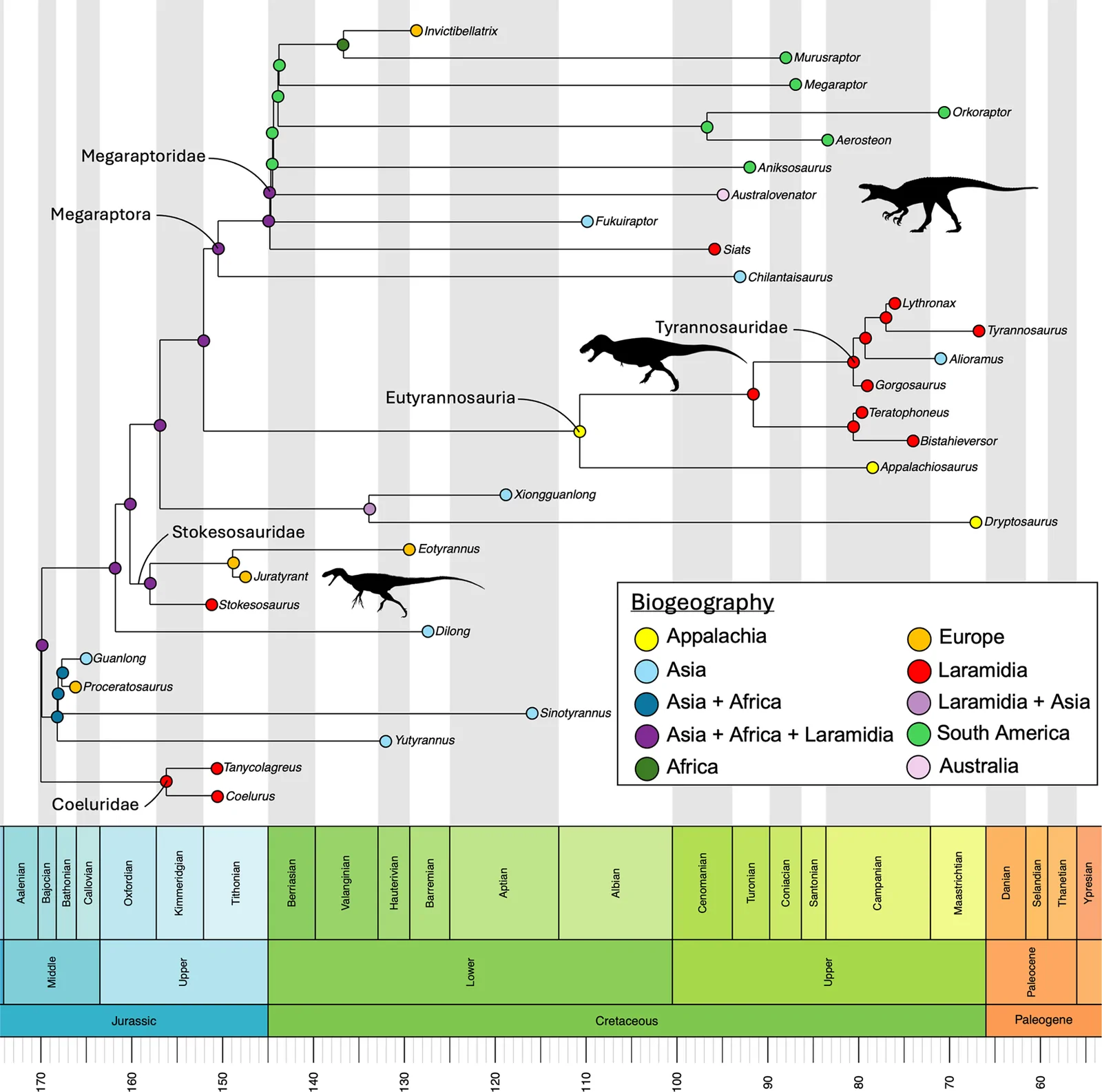
Time-calibrated strict consensus tree based on the Naish & Cau [24] data matrix under extended implied weights and biogeographical results of Tyrannosauroidea, including Megaraptora and *I. amberina*, including likely geographical origins. Lara. + app. refers to Laramidia and Appalachia. Silhouettes from Phylopic: *A. wintonensis* by T. Tischler used under the License Attribution-ShareAlike 3.0 unported https://creativecommons.org/licenses/by-sa/3.0/; *T. rex* by Scott Hartman used under the Attribution-NonCommercial-ShareAlike 3.0 unported https://creativecommons.org/licenses/by-nc-sa/3.0/ and *S. clevelandi* by Scott Hartman (modified by T. Michael Keesey) used under the Attribution-NonCommercial-ShareAlike 3.0 unported https://creativecommons.org/licenses/by-nc-sa/3.0/.

### 3.6. Phylogenetic analysis: Naish & Cau [24] matrix

Analysis with equal weighting (EQW) yields 36 most parsimonious trees (MPTs) with lengths of 4411 steps (consistency index (CI) = 0.304; retention index (RI) = 0.522), with a single MPT of length 199.34 steps resulting from analysis with extended implied weights (EIW) (CI = 0.303; RI = 0.519). The two analytical approaches produce broadly congruent topologies. In both analyses, *I. amberina* is recovered as the sister taxon of *Murusraptor*, with this clade within late-diverging Megaraptoridae (figures 5 and 6). The relationships within Megaraptora differ between the two analyses (figures 5 and 6). For example, in the EIW analysis (figure 6), *Australovenator* is recovered as a non-megaraptorid megaraptoran and *Aniksosaurus* is recovered as a megaraptorid, whereas in the EQW analysis (figure 5) the opposite is true. Under EQW, Megaraptora has a Bremer support of 2, whereas Megaraptoridae and the sub-clade *Murusraptor* + *Invictibellatrix* each have support of 1.

The EQW analysis recovers seven synapomorphies for Megaraptora: (i) caudal vertebrae, chevrons, middle elements and anteroventral process: absent, ‘L’-shaped chevrons (C354 [0]), (ii) caudal vertebrae, anterior neural arches and ventral rib laminae: present (C358 [1]), (iii) ilium and lateral vertical crest dorsal to the acetabulum: absent (C382 [0]), (iv) vertebrae, pleurocoels and shape: slit-like, dorsoventrally narrow (C847 [1]), (v) dorsal vertebrae, anterior neural spines and inclination: anterodorsally directed (C851[1]), (vi) dorsal vertebrae, anterior neural arches, base of prezygapophyses and pneumatic recesses: present (C942 [1]), and (vii) dorsal vertebrae, anterior and middle neural arches, anterior infraprezygapophyseal fossae and development: deep (C1235 [1]). In this analysis, Megaraptoridae is supported by four synapomorphies: (i) manual ungual I, flexor tubercle, ventral surface and transverse groove: present (C1062 [1]), (ii) ulna, proximal third of shaft, anterior margin and thick ridge from coronoid (anterior/sigmoid) process: present (C1108 [1]), (3) coracoid and lateral fossa ventrodistal to glenoid (sub-glenoid fossa): present (C1173 [1]), and (4) ulna, olecranon process and shape in lateral/medial view: trapezoidal and distally tapering (C1593[1]).

The EIW analysis recovers four synapomorphies for Megaraptora: (i) ilium, pubic peduncle, distal surface and anteroposterior length: less than two times its mediolateral width (C396 [0]), (ii) tibia, anterodistal end and proximodistally elongate medial crest: present (C578 [1]), (iii) ilium, preacetabular process, ventral surface and posterior end: mediolaterally expanded (C1549 [1]), and (iv) humerus, deltopectoral crest, proximoanterior margin and shape in medial/lateral view: concave and crest abruptly projected distal to head (C1600 [1]). Megaraptoridae is recovered with three synapomorphies: (i) postaxial cervical prezygoepipophyseal lamina: present (C209 [1]); (ii) caudal vertebrae, centra and pleurocoels: present (C372 [1]); and (iii) caudal vertebrae, anterior neural arches and pneumatic recesses: present (C1185 [1]).

Under both EQW and EIW analyses, *Murusraptor* and *Invictibellatrix* are recovered in a clade supported by two synapomorphies: (ii) foramen magnum and shape: elliptical and taller dorsoventrally than mediolaterally wider with a dorsoventral height that exceeds mediolateral width (C112 [1]), and (ii) basal tubera, mediolateral width between the paired processes and less than the mediolateral width of the occipital condyle (C117 [1]).

### 3.7. Phylogenetic analysis: Aranciaga Rolando *et al.* [29] matrix

Analysis of the Aranciaga Rolando *et al.* [29] matrix using EQW produces 47 MPTs with lengths of 1437 steps (CI = 0.315, RI = 0.597). The strict consensus of these recovers *Invictibellatrix* as a megaraptoran, later branching than *Phuwiangvenator*; a large polytomy exists between all taxa later-branching than *Phuwiangvenator*. Using EIW recovers 23 MPTs with lengths of 76.91562. The same topology within Megaraptora is recovered. Our FBD model resolves the polytomy within Megaraptora and posits *Invictibellatrix* as the sister taxon to *Fukuiraptor* + Megaraptoridae (figure 7).

**Figure 7.**
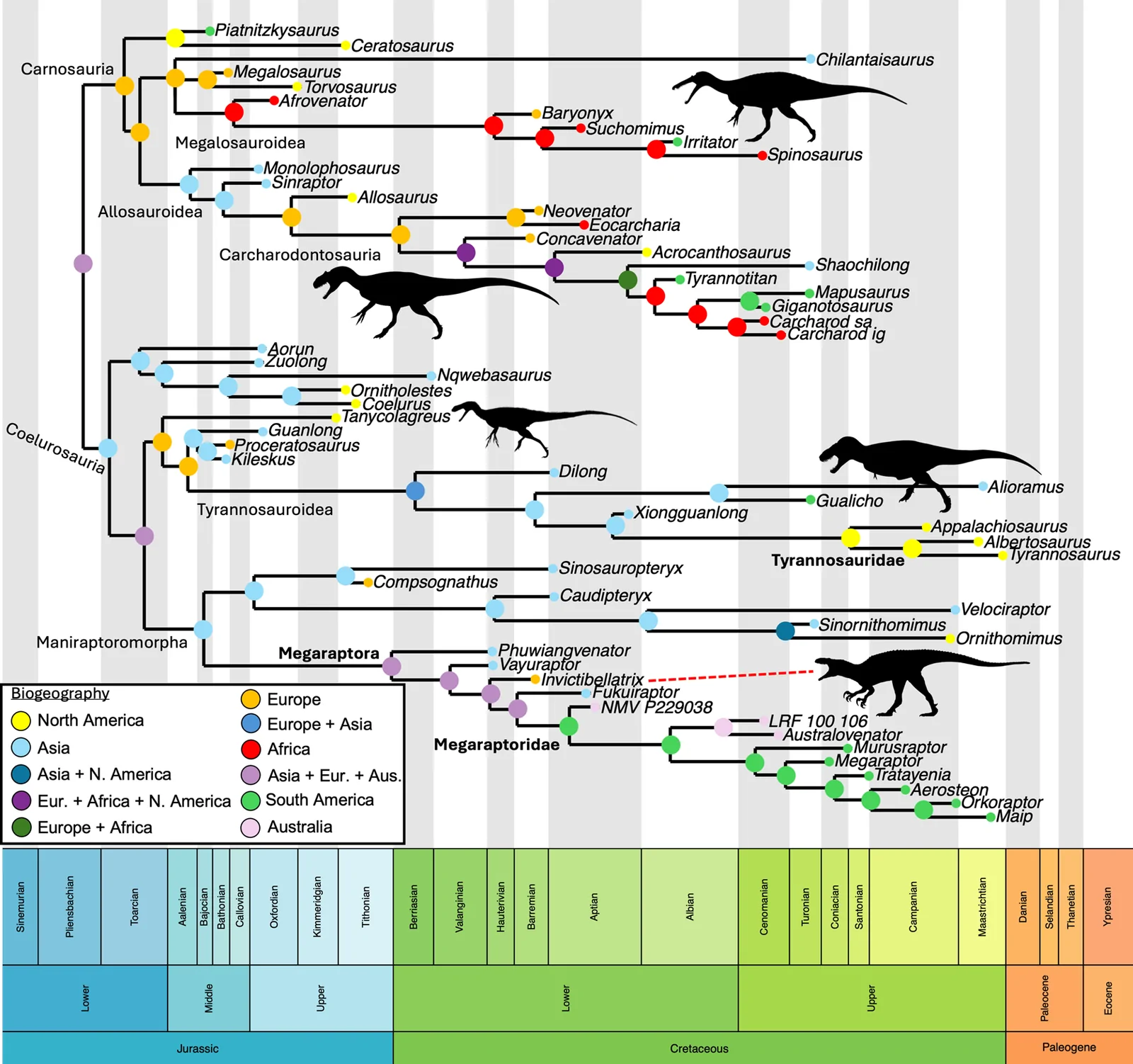
Time-calibrated strict consensus tree based on the Aranciaga Rolando *et al.* [29] data matrix using a FBD analysis, including the new taxon, *I. amberina*. Silhouettes from Phylopic: *Cristatusaurus lapparenti* by Scott Hartman used under the Attribution-NonCommercial-ShareAlike 3.0 unported https://creativecommons.org/licenses/by-nc-sa/3.0/; *A. fragilis* by Scott Hartman used under the Attribution-NonCommercial-ShareAlike 3.0 unported https://creativecommons.org/licenses/by-nc-sa/3.0/; *S. clevelandi* by Scott Hartman (modified by T. Michael Keesey) used under the Attribution-NonCommercial-ShareAlike 3.0 unported https://creativecommons.org/licenses/by-nc-sa/3.0/; *T. rex* by Scott Hartman used under the Attribution-NonCommercial-ShareAlike 3.0 unported https://creativecommons.org/licenses/by-nc-sa/3.0/ and *A. wintonensis* by T. Tischler used under the license Attribution-ShareAlike 3.0 unported https://creativecommons.org/licenses/by-sa/3.0/.

Both the EQW and EIW analyses recover four synapomorphies for Megaraptora: (i) manual ungual I-2, transverse compression: strongly transversely compressed and being oval with a distinct dorsoventral axis in proximal view (C141 [1]); (2) astragalus, with a prominent posterolateral extension: present (C198 [1]); (iii) sacrum and ventral surface: flattened and usually with a ventral sulcus (C214 [1]); and (4) tibia and direction of the cnemial crest: anteriorly directed (C354 [1]).

Under both EQW and EIW analyses, *Murusraptor* and *Invictibellatrix* are recovered in a clade supported by one synapomorphy: braincase, angle between the main axis of the occipital condyle and the transverse section of basal tubera: less than 75°, occipital surface sloping posteroventrally (C68 [1]). There are likely three local/shared synapomorphies of Megaraptora that *Invictibellatrix* possesses: (i) braincase and pneumatization: highly pneumatic (C69 [2]), (ii) occipital condyle and contour: dorsoventrally compressed (C77 [1]), and (iii) basisphenoid and fossa deep and funnel-shaped: present (C78 [1]).

### 3.8. Biogeographical results

Our biogeographic analyses using the trees derived from the updated Naish & Cau [24] data matrix identify the BAYAREALIKE+J model as the best fit to the data for both the EQW- and EIW-based topologies, with 81–94% of the data being explained by this model (table 4). This model selection indicates that theropod biogeography is driven by widespread sympatry, widespread distribution of ancestral stocks, founder events and regional extinction resulting in geographic range contraction. All models, including a jump dispersal parameter, are preferred to those which do not include this (*P*-value << 0.01; table 1). Other biogeographical models (DEC+J, DIVALIKE+J) have minimal support in our analyses, with DEC+J explaining only 16% of the data analysed. This suggests that vicariance did not play a large role in shaping tyrannosauroid biogeography. Ancestral area reconstructions suggest that megaraptorans were widespread, inhabiting Laramidia, Asia and Africa during the Early Cretaceous (approx. 145–138 Ma), and an African population dispersed to Australia via South America and Antarctica during this interval (figures 5 and 6). *Invictibellatrix* is inferred to have dispersed into Europe from an African, Asian or South American megaraptoran population approximately 130 Ma, the same population which gave rise to the *Murusraptor* + *Megaraptor* clade, indicating that derived megaraptorids were widespread early on in their evolutionary history (figures 5 and 6).

**Table 4.** Results of the BioGeoBEARS analyses using the topologies generated from the modified Naish & Cau [24] data matrix.

BioGeoBEARS		AICc analysis		likelihood analysis		
analysis	model	AICc	AICc wt%	LnL	D-statistic	P-Value
**EQW**	DEC	232.7	4.4*10^–8^	–114.1		
	DEC+J	209	6.3*10^–3^	–101	26.22	3*10^–7^
	DIVALIKE	258.9	9.2*10^–16^	–127.2		
	DIVALIKE+J	198.2	1.3	–95.66	63.11	2*10^–15^
	BAYAREALIKE	195.6	4.9	–96.6		
	BAYAREALIKE+J	**189.8**	**94.0**	**–91.42**	8.37	0.0038
**EIW**	DEC	196.8	2.7*10^–3^	–96.19		
	DEC+J	179.4	16	–86.24	19.9	8.1*10^–6^
	DIVALIKE	221.2	1.4*10^–8^	–108.4		
	DIVALIKE+J	201.7	2.4*10^–4^	–97.39	21.97	2.8*10^–6^
	BAYAREALIKE	182.8	3.0	–89.18		
	BAYAREALIKE+J	**176.2**	**81.0**	**–84.64**	9.09	0.0026

By contrast, analysis of the Aranciaga Rolando *et al.* [29] FBD topology results in the DEC+J model being recovered as the best fit to the available data (table 5). This suggests that vicariance played a significant role in the evolution of the sampled theropods and, particularly, Megaraptora. Other models are substantially less well-supported by our analyses. Ancestral area reconstructions of this topology suggest a different dispersal route for megaraptorans. The earliest Cretaceous of Asia is inferred as the area of origin for Megaraptora, with *Invictibellatrix* demonstrating that dispersal throughout Eurasia occurred by the Barremian. Dispersal through Europe and into Africa is implied by the presence of a contiguous palaeobiogeographic corridor between Europe, Africa and South America during the Early Cretaceous [111,112]. Megaraptoridae is inferred to have originated in South America, with Australian taxa forming a clade sister to all South American megaraptorids (figure 7).

**Table 5.** Results of the BioGeoBEARS analyses of the Aranciaga Rolando *et al.* [29] FBD topology.

BioGeoBEARS		AICc analysis		likelihood analysis		
**analysis**	**model**	**AICc**	**AICc wt%**	**LnL**	**D-statistic**	***P*-Value**
**relaxed**	DEC	274.4	1.1e-7	–135.1		
	**DEC+J**	**243.4**	**0.61**	**–118.5**	**33.31**	**7.9e-9**
	DIVALIKE	266.1	6.9e-6	–131		
	DIVALIKE+J	244.2	0.39	–118.9	24.13	9e-7
	BAYAREALIKE	327.4	3.5e-19	–161.6		
	BAYAREALIKE+J	265.8	8.2e-6	–129.7	63.81	1.4e-15
**harsh**	DEC	286.3	0.0006	–141		
	**DEC+J**	**271.4**	**1**	**–132.5**	**17.18**	**3.4e-5**
	DIVALIKE	330.7	1.3e-13	–163.3		
	DIVALIKE+J	310.3	3.5e-9	–151.9	22.65	1.9e-6
	BAYAREALIKE	340.7	8.8e-16	–168.2		
	BAYAREALIKE+J	325	2.3e-12	–159.3	17.91	2.3e-5

## 4. Discussion

### 4.1. Megaraptoran phylogenetic placement and terminology

Our phylogenetic analyses, as well as those of prior studies, recover Megaraptora within Tyrannosauroidea, while others continue to find support for alternative phylogenetic placements (§1). Therefore, although we find support for a tyrannosauroid affinity, we refer to *Invictibellatrix* and its close relatives as megaraptorans, and refrain from describing them as ‘megaraptoran tyrannosauroids’. Although this would be logical given our taxonomic hypothesis, it fails to highlight the phylogenetic uncertainty regarding the taxonomic affinities of Megaraptora among tetanurans.

### 4.2. Comparisons with other theropod clades

The only theropod taxa from the Wessex Formation that preserve overlapping material with *Invictibellatrix* are the spinosaurids, *Riparovenator* and *Ceratosuchops* [12], alongside the potential maniraptoran *Yaverlandia* that is known only from a partial skull roof [16]. In addition to these three species, we can make anatomical comparisons with other representatives of the theropod clades present in the Wessex Formation, namely: Spinosauridae, Tyrannosauroidea, Carcharodontosauridae, Maniraptoriformes and Noasauridae, although the presence of the latter is disputed [17,18]. The degree of fusion of the neurocranial bones within the skull of the *Invictibellatrix* holotype indicates that it is unlikely to be a juvenile or sub-adult individual, and thus, we consider the possibility that ontogenetic stage might render our interpretation of its taxonomic identification unlikely.

The only known noasaurid braincase is that of *Masiakasaurus* ([90]; FMNH PR 2457), but it is incomplete, limiting comparisons with *Invictibellatrix*. However, some details can be compared between these taxa. The shape of the occipital condyle in *Masiakasaurus* is strongly dorsoventrally compressed compared with the preserved portion of the foramen magnum (less than half the dorsoventral height of the latter), whereas the condyle of *Invictibellatrix* is less compressed (greater than half the dorsoventral height of the foramen magnum). In addition, the basisphenoid recess is teardrop-shaped in *Masiakasaurus*, whereas it is sub-circular in *Invictibellatrix* (and many other theropods) [90], differentiating the latter from the only known noasaurid braincase. Furthermore, the proportions of the braincase ventral to the occipital condyle relative to the rest of the dorsoventral height of the braincase differ between *Masiakasaurus* and *Invictibellatrix*. In the former, the portion below the condyle is much shorter dorsoventrally compared with the latter, suggesting a taller braincase in *Invictibellatrix*. Additionally, the paracondylar recesses of *Invictibellatrix* are transversely narrower than those in *Masiakasaurus*, further differentiating the two. The braincase of *Invictibellatrix* is also less extensively pneumatized than that of *Masiakasaurus*, with the respective fossae (such as the posterior tympanic recess) being much deeper in the latter taxon. It is therefore clear that *Invictibellatrix* does not represent a member of Noasauridae.

The braincase of *Invictibellatrix* does not display any substantial similarities to those of spinosaurids, including *Riparovenator*, *Ceratosuchops* [12] and *Baryonyx* [25]. Spinosaurid braincases are unique among theropods in being taller dorsoventrally than they are anteroposteriorly long [12,25,95], differing substantially from the proportions of *Invictibellatrix*. Further differences between spinosaurids and *Invictibellatrix* include: basisphenoid recess of *Invictibellatrix* is not slit-like as in spinosaurids, but is = anteroposteriorly expanded and rounded in ventral view; in *Invictibellatrix* the basipterygoid processes and basal tubera are widely separated and the former are not ventral to the latter; and the foramen for CN VII is not dorsoventrally expanded in *Invictibellatrix*, contrasting with the condition in *Ceratosuchops* and *Baryonyx* ([113]; pers. obvs.). Owing to the highly characteristic nature of spinosaurid braincases, the absence of these features in *Invictibellatrix*, and the results of our phylogenetic analyses, we consider it highly unlikely that the new taxon can be referred to Spinosauridae.

*Invictibellatrix* lacks many carcharodontosaurid synapomorphies [74]: (i) the opening for CN V is ventral to the nuchal crest in *Invictibellatrix*, whereas this opening is posterior to the crest in Carcharodontosauridae, (ii) the ventromedial region of the paraoccipital process in *Invictibellatrix* lacks a large open area exposing the inner ear region in posterior view, whereas this is exposed in Carcharodontosauridae, and (iii) the neck of the occipital condyle of *Invictibellatrix* lacks pneumatic cavities that join medially in the condylar neck, whereas these are present in Carcharodontosauridae. Furthermore, *Invictibellatrix* lacks two synapomorphies of Allosauroidea: (i) a fully split trigeminal nerve (CN V; [114,115]), as only a single opening for CN V is present on the lateral surface of the braincase, and (ii) the supraoccipital knob being substantially wider than the foramen magnum [115,116] as the knob is only as wide as foramen magnum in *Invictibellatrix* (figure 3). This indicates that *Invictibellatrix* cannot be referred to as Allosauroidea. As such, we do not consider it likely that *Invictibellatrix* represents a member of Carcharodontosauridae, nor Allosauroidea. Currently, *Neovenator* is the only named carcharodontosaurian/allosauroid known from the Wessex Formation [45]; although it does not preserve overlapping elements with *Invictibellatrix*, the foregoing comparisons suggest that it is highly unlikely that the latter would be a junior synonym of the former.

It is possible that the holotype of *Invictibellatrix* represents a tyrannosauroid (if excluding megaraptorans), potentially an additional specimen of *Eotyrannus*. However, numerous features present in *Invictibellatrix* contrast strongly with those of tyrannosauroids, such as *Timurlengia euotica* [52] and *Xiongguanlong* [53], which help preclude referral to an early-diverging tyrannosauroid (such as *Eotyrannus*; *sensu* [52,117]). These include: (i) a strong mid-brain flexure in *Invictibellatrix* (weak in tyrannosauroids; [52,118]), (ii) basal tubera taller than the occipital condyle (whereas the basal tubera are shorter than the condyle in early tyrannosauroids; [52,54,55]), (iii) a relatively shallow posterior tympanic recess (versus a deep and invasive recess in tyrannosauroids), (iv) presence of a sub-condylar recess (absent in early tyrannosauroids; [52,53,55]), and (v) deep paracondylar recesses (which are shallow in early tyrannosauroids; [55]). Although some features do align with early tyrannosauroids, such as the presence of a midline ridge (=parasagittal nuchal crest) on the posterior surface of the supraoccipital, the combination and development of the suite of features recognized in *Invictibellatrix* indicate that it can be referred to a non-maniraptoriform coelurosaur but not to an early-diverging tyrannosauroid (sensu [52]). It should be noted, however, that *Eotyrannus* likely occupies a much earlier-diverging position within tyrannosauroid evolutionary history and so would not be expected to share all the features seen in ‘mid-grade tyrannosauroids’, such as *Timurlengia* or *Xiongguanlong*. Despite this, the absence of numerous purported tyrannosauroid symplesiomorphies currently precludes referral of our specimen to Tyrannosauroidea.

The braincases of maniraptoriforms are highly derived and possess features, such as an expanded hindbrain, low angles between the hind- and midbrain [119–121], and a foramen magnum that is wider than the occipital condyle [60]. The hindbrain of *Invictibellatrix* is weakly expanded, and the braincase has a narrower foramen magnum than the occipital condyle. Furthermore, our phylogenetic analyses do not recover *Invictibellatrix* as part of Maniraptoriformes. Therefore, we rule out maniraptoriform identification for *Invictibellatrix*.

Analyses of both Naish & Cau [24] and Aranciaga Rolando *et al.* [29] matrices recover *Invictibellatrix* within Megaraptora. The phylogenetic position of Megaraptora is controversial as recent analyses recover the clade either in a sister-group relationship with Tyrannosauroidea [29] or within Tyrannosauroidea [24,46], whereas earlier studies found them to be earlier-branching coelurosaurians [122] or later-diverging allosauroids ([45]). However, although the placement of Megaraptora is unresolved, members of the clade can be distinguished from tyrannosauroids based on numerous cranial and postcranial characters [18,24,29]. While the most recent phylogenetic analyses consistently recover Megaraptora as being closely related to Tyrannosauroidea, it should be noted that no sufficiently complete megaraptoran skulls are currently known. The skull of Megaraptor is the best preserved but it belongs to a juvenile individual, potentially imparting an ontogenetic bias, as significant morphological variation has been observed in other tyrannosauroids [75,123–127]. The only other non-maniraptoriform coelurosaurian known currently from the Wessex Formation is *Eotyrannus*, but there is no overlapping material with *Invictibellatrix*. *Eotyrannus* has consistently been recovered as a tyrannosauroid, although its placement within the clade has varied between an early-diverging tyrannosauroid [97], stokesosaurid [128,129], proceratosaurid [130] and a megaraptoran. A recent analysis based on a detailed monographic description recovered *Eotyrannus* as a stokesosaurid, just outside of the tyrannosaurid radiation [24], which is also supported by our EIW analysis (figure 6), whereas it is recovered as an early-diverging tyrannosauroid in our EQW analysis (figure 5). The combination of occipital condyle shape, foramen magnum shape and FOR value enables megaraptorans, tyrannosaurids and proceratosaurids to be distinguished from one another. Moreover, *Invictibellatrix* is characterized by several braincase characters otherwise seen primarily in megaraptorans, and as such are likely local/shared synapomorphies of the clade (C112 [1] and C117 [1] [24]; C69 [2], C77[1] and C78[1] [29]: see above for details). Additional analyses using the updated Ibiricu *et al.* [65] matrix recover *Invictibellatrix* within Megaraptora (supplementary material, figures S1 and S2). Thus, regardless of the phylogenetic placement of Megaraptora within Theropoda and in concert with the anatomy of *Invictibellatrix*, we propose that this taxon is a member of Megaraptora and is not synonymous with other named Wealden theropods.

### 4.3. The potential of endocranial characters for reconstructing theropod phylogeny

Braincase characters are usually limited in number within phylogenetic analyses, even in the most recent and largest theropod phylogenetic datasets. For example, Naish & Cau [24] included 1759 characters, but only 74 of these are braincase characters, accounting for 4.2% of those available. Within the Aranciaga Rolando *et al.* [29] matrix 3.9% of characters relate to the braincase (14 of 357 characters). The most recent version of the ‘Theropod Working Group Matrix’, which is dominated by coelurosaurian taxa, currently has 841 characters, of which 94 pertain to the braincase, accounting for 11.1% of those available [131]. This is perhaps surprising, as the braincase contains multiple bones, has complex morphology and can now be studied in greater detail owing to new technologies that enable the development of detailed digital reconstructions (e.g. [31]). However, to date, there has been little published work on theropod braincases that has attempted to identify features that could be used in phylogenetic analyses. This is probably owing to the widely held assumption that neurocrania are anatomically conservative [113], but, as shown here, there is phylogenetic variation in at least some braincase characters that could help to resolve the interrelationships of theropods, especially for groups with uncertain or conflicting placements (or that are based on limited material). Further detailed work to document the variation in theropod braincases might yield additional phylogenetically informative features.

### 4.4. Phylogenetic implications of the foramen magnum width to occipital condyle width ratio

There are some general trends in the foramen magnum width to occipital condyle width ratios (FOR) of theropods, with early-diverging theropods having a FOR greater than 1, FOR decreasing to less than 1 in early tetanuran theropods and then increasing above 1 in Noasauridae and derived members of Coelurosauria (figures 8 and 9). While there is overlap, certain clades such as Megaraptora, Allosauroidea and Megalosauroidea take up disparate but overlapping regions in morphospace (table 3). Megaraptora (0.68–0.88) and Megalosauroidea (0.65–0.83) occupy a ‘middle range’ of FOR values. Abelisaurids have a FOR range of 0.56–0.82, carcharodontosaurids 0.36-0.53, and tyrannosaurids 0.27–0.59, illustrating that the FOR range can help to differentiate theropod clades owing to the ratios having some distinction between theropod clades.

**Figure 8.**
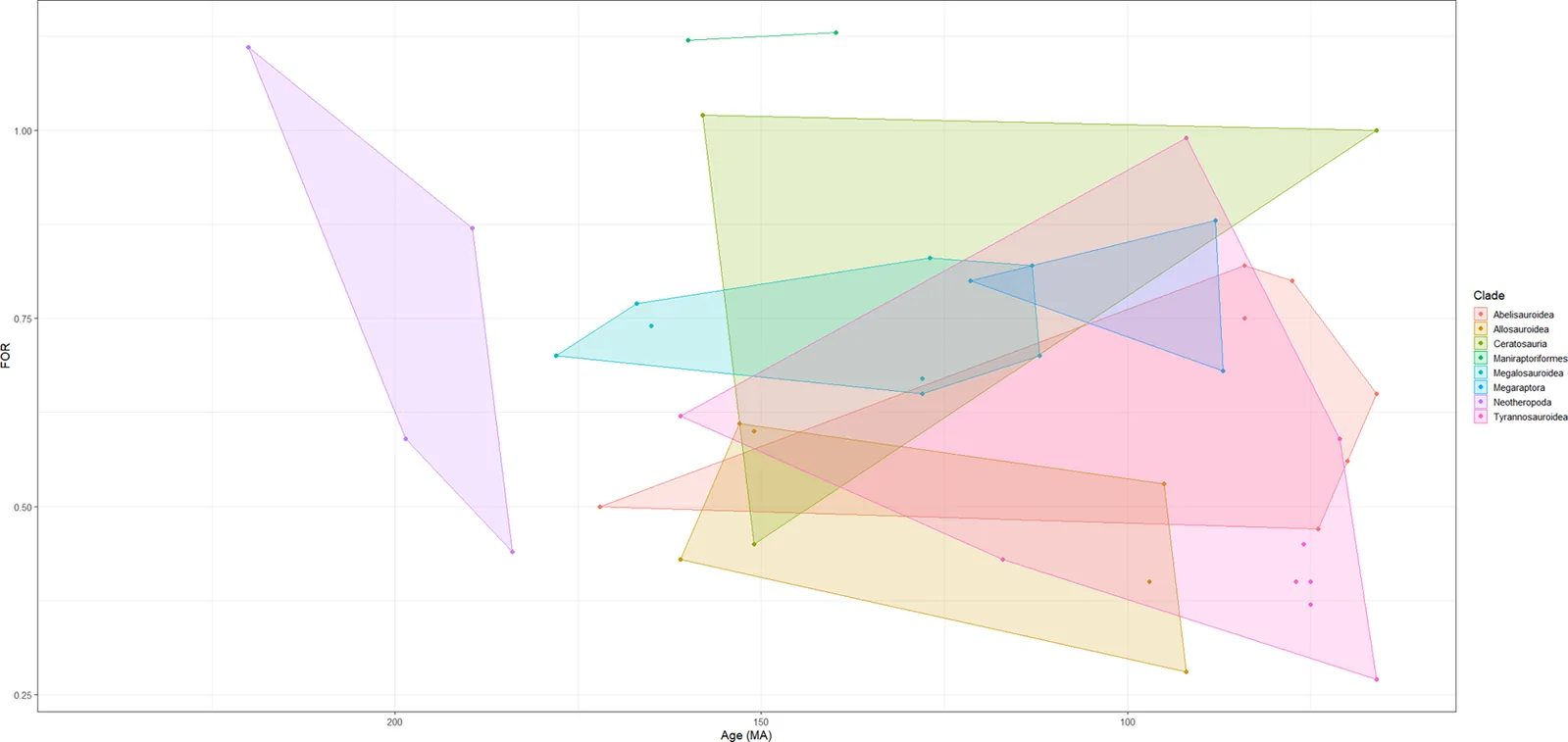
The FOR of the different theropods plotted as clades throughout the Mesozoic based on the values from table 3.

**Figure 9.**
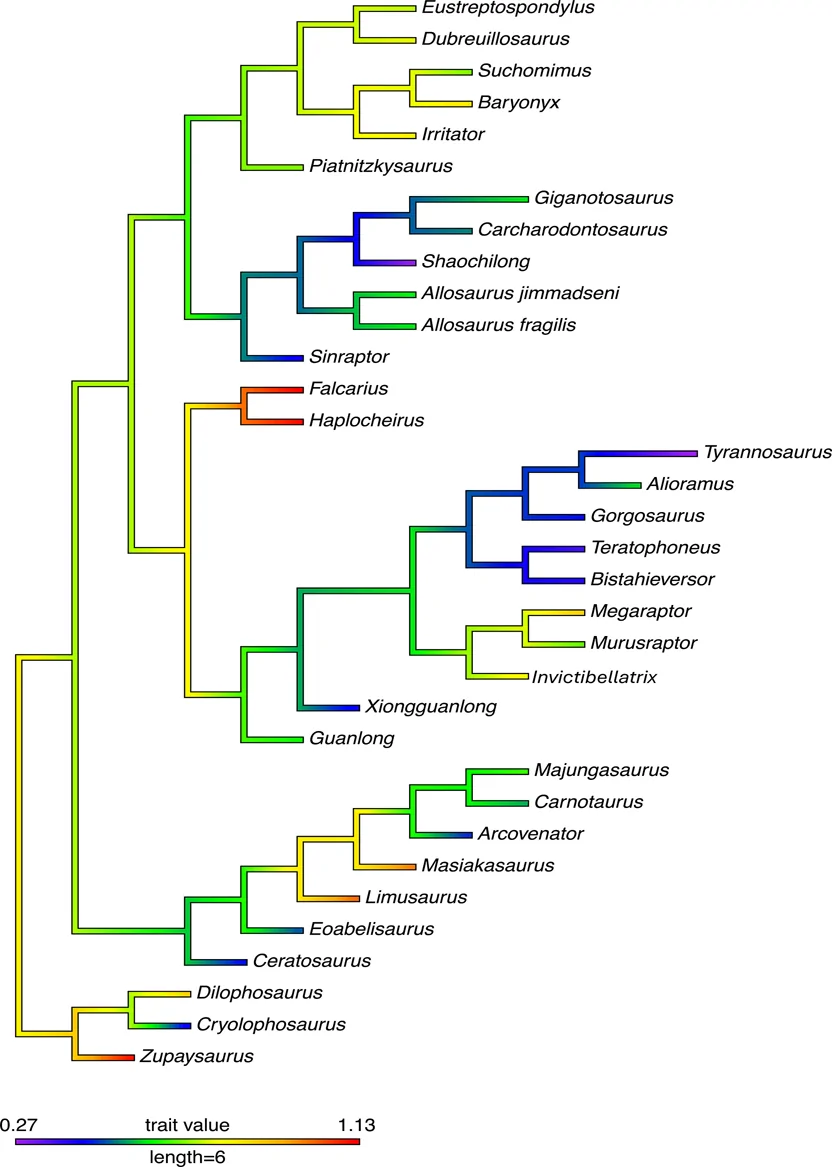
Phylogenetic plot of the FOR with warmer colours representing an increased FOR and cooler colours representing a lower FOR across neotheropods based on the FOR values in table 3 and using the EQW phylogeny derived from the modified Naish & Cau [24] data matrix.

### 4.5. Megaraptoran biogeography

*Invictibellatrix amberina* represents the first megaraptoran to be described from Europe and is of comparable age with other Laurasian non-megaraptorid megaraptorans, including *Fukuiraptor* (Barremian–Aptian Kitadani Formation, Japan) and *Phuwiangvenator* (upper Hauterivian to lower Valanginian Sao Khua Formation, Thailand) [67,132,133], providing insight into the early biogeographical history of this clade. Biogeographic analysis of the Naish & Cau [24] topology suggests that vicariance did not play a prominent role in shaping tyrannosauroid biogeography (but see [39]). Overall, our analyses are ambiguous as to the biogeographic origins of *Invictibellatrix*, with Africa + Asia + South America (figures 5 and 6) and Eurasia + Australia (figure 7) ancestral areas estimated for ancestral nodes for this species. However, the Naish and Cau [24] topology includes numerous disputed megaraptoran taxa (*Siats*, *Aniksosaurus* and *Chilantaisaurus*), which may have negatively influenced the analysis and added biogeographic noise into the analyses. Furthermore, the Aranciaga Rolando *et al.* [29] dataset includes a greater sampling of putative megaraptorans, suggesting greater resolution and accuracy than the Naish & Cau [24] topology. Indeed, this topology provides more detailed insight into megaraptoran biogeography, and it also suggests that megaraptorans were widely distributed early in their evolutionary history (see also [39]). As a result, we prefer the insights provided by the Aranciaga Rolando *et al.* [29] topology (figure 7). Therefore, we infer that *Invictibellatrix* likely evolved from an Asian megaraptoran population that dispersed into Europe approximately 130 Mya, supporting recent work which found a widespread distribution during the Early Cretaceous [39]. This was followed by subsequent megaraptoran dispersal to Africa and Australia, before giving rise to megaraptorids. This is implied by the very large ancestral area estimates including multiple relatively distant landmasses (e.g. Asia + Australia) and the most parsimonious dispersal route into Gondwana occurring via Europe into Africa.

Our results further indicate a widespread ancestral distribution of later-diverging megaraptorans, being present in Africa, Asia and South America. A cosmopolitan early distribution that includes Gondwana is implied by the presence of the megaraptorid *Australovenator* in the Cenomanian of Australia [134,135]), while the oldest Gondwanan megaraptoran is currently from the Albian of South America [136]. Sampling bias likely explains the scarcity of megaraptorans elsewhere in Gondwana during the Early Cretaceous [18,66]. Subsequent dispersal into Africa is also supported by our results as they suggest that Megaraptoridae originated in western Gondwana (South America + Africa) and dispersed into Australia. This conflicts with previous work suggesting that South American megaraptorids arose from an Australian population [65,66]. This does, however, support the hypothesis of a late acquisition of larger body size in the clade [39] as *Invictibellatrix* is likely among the smallest megaraptorans (<4 m length) discovered to date.

The presence of closely related contemporaneous taxa in Africa and Europe in the Early Cretaceous, such as the spinosaurids *Baryonyx* and *Suchomimus*, and the rebbachisaurid sauropods *Demandasaurus* and *Nigersaurus* (Torcida [12,137]), suggests either faunal exchange between the two regions and/or the widespread distribution of a cosmopolitan fauna [135], with *Invictibellatrix* contributing to this assessment. Subsequent regional extinctions likely resulted in range contractions, leading to the observed geographic range in the Late Cretaceous (figures 5 and 6). There is evidence for the extirpation of Megaraptora in Laurasia, along with Allosauroidea, in the early Late Cretaceous [57], whereas megaraptorans continued to flourish and diversify in the Late Cretaceous of Gondwana [18,30,39].

### 4.6. Theropod faunal assemblages within the Wessex Formation

The Wessex Formation spans 15 million years, and recent studies of its sauropod and ornithischian faunal assemblages show a much higher species richness than previously recognized (e.g. [5,7,15,138–140]). Although its exact stratigraphical position is unknown, the holotype of *Invictibellatrix* was most likely recovered from the lower plant debris beds (L1–5), as these beds outcrop at Sudmoor Point from where the holotype specimen was recovered [27]. As the first report of a megaraptoran from the formation, *Invictibellatrix* adds to the already diverse but fragmentary theropod faunal assemblage (figures 10 and 11; [6]).

**Figure 10.**
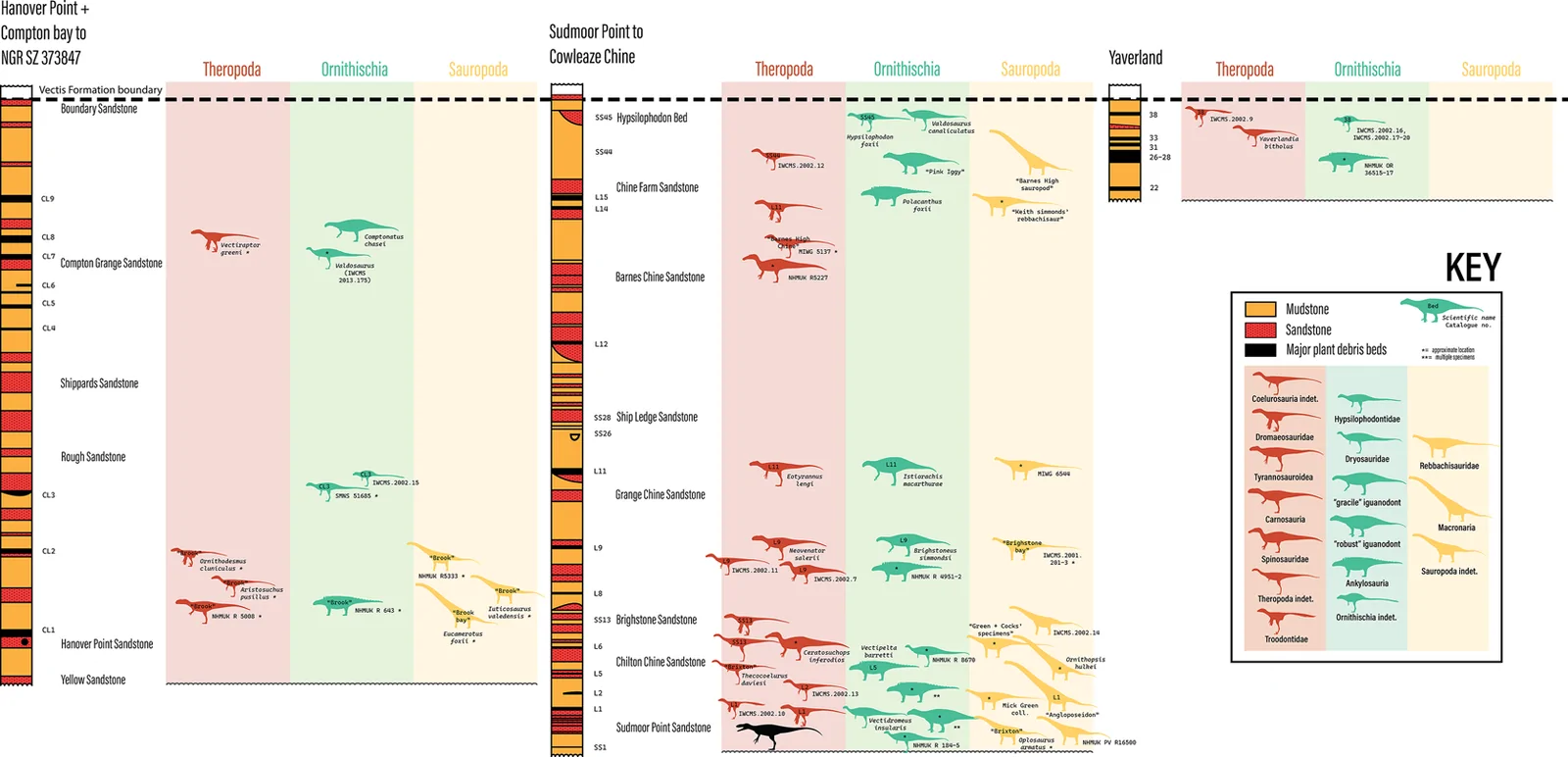
Stratigraphical distribution of known dinosaurian fauna across the Wessex Formation spread, split into the three dinosaurian clades: theropods, ornithischians and sauropods to determine faunal assemblage diversity and sympatry across the Wessex Formation (see supplementary material for full details of taxa locations). The black silhouette represents the stratigraphical location of *Invictibellatrix*. Silhouettes by D.D.F.

**Figure 11.**
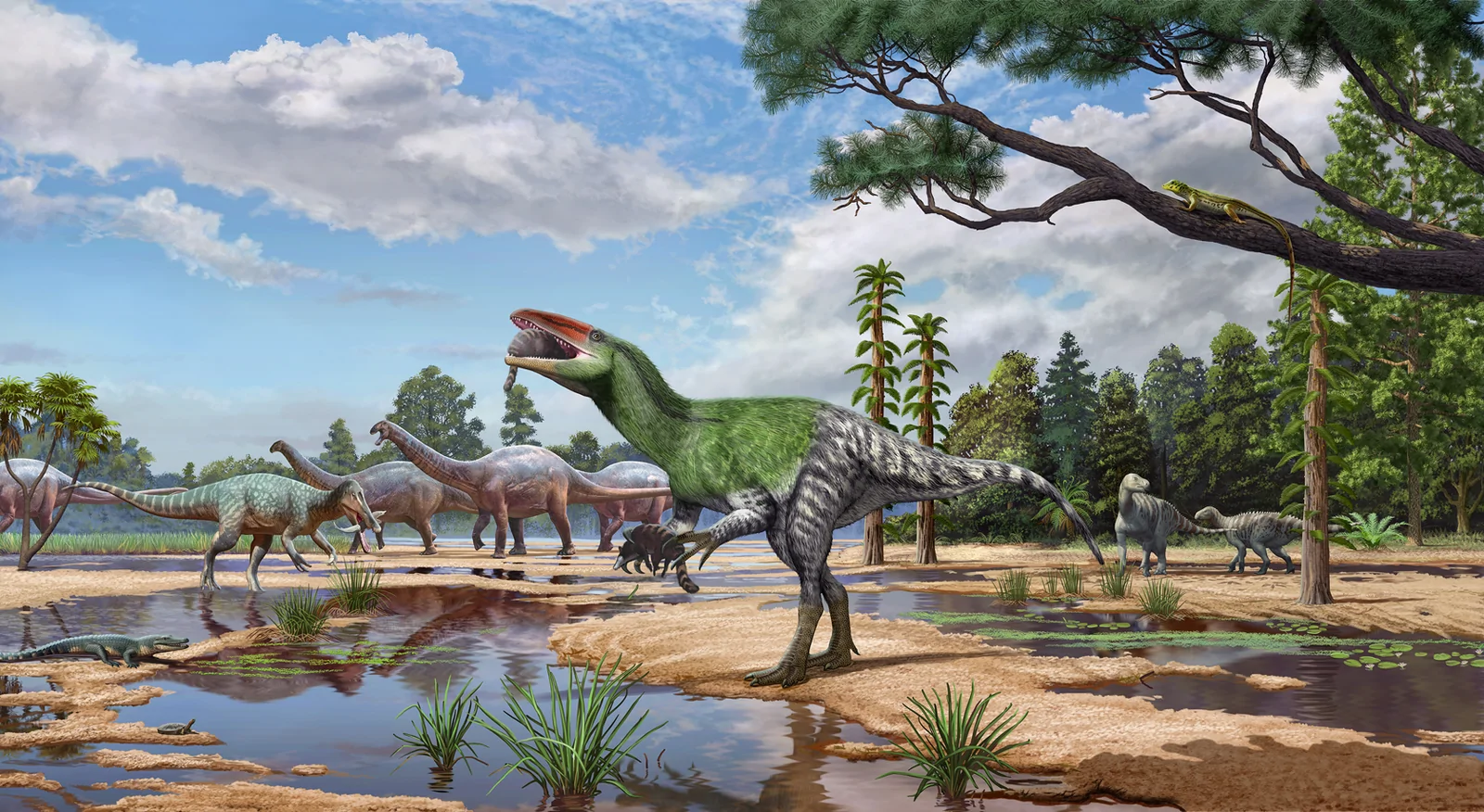
Artists’ rendition of *I. amberina* with contemporary fauna in the lower part of the Wessex Formation. By Sergey Krasovskiy and Pedro Salas.

The observed taxonomic diversity of theropods varies throughout the Wessex Formation, with the greatest diversity from the stratigraphically oldest parts of the formation (from plant debris beds L1–8), with a slight decrease from plant debris bed L9 upwards, and then an increase in diversity from L14 until the boundary with the overlying Vectis Formation (figure 10). These changes correlate with changes in the depositional environment, from lowland/coastal habitats in L1–8 (figure 11), to more upland environments in L9, returning to coastal/lowland environments until the boundary with the overlying Vectis Formation. Fluctuations in the taxonomic diversity of Wessex Formation theropods might therefore relate to the proximity to water or increased preservation potential of coastal/lowland environments in a heterogeneous mosaic environment [2,27,141].

## 5. Conclusions

*Invictibellatrix amberina* gen. et sp. nov. is a new non-megaraptorid megaraptoran from the Lower Cretaceous Wessex Formation of the UK and the first from Europe. This discovery supports the view that Megaraptora was a near-globally distributed clade early in its evolutionary history. *Invictibellatrix* likely evolved from an Asian megaraptoran population, before dispersing into European biotas. These European megaraptorans then dispersed through Africa and the rest of Gondwana, with sampling failure the likely explanation for the group's interpreted absence from the Early Cretaceous Gondwanan fossil record. Regional extinctions of megaraptorans in the Late Cretaceous of Laurasia led to the observed range contraction of the group, with only Gondwanan megaraptorids surviving. Finally, we demonstrate that braincase characters, which are under-represented in theropod phylogenetic analyses, have the potential to help illuminate the relationships between theropod clades, including shedding light on the problematic position of Megaraptora.

## Supplementary Material



## Data Availability

The specimen NHMUK PV 6775 is housed at the NHMUK for other researchers to access. The character matrix scoring (in the form of a TNT file), discussion of the other character changes and the biogeographic files are available as part of the supplementary information [142]. The CT scans for NHMUK PV 6775 are here on MorphoSource: https://www.morphosource.org/media-lists/000904827.
