# Supplementary material for: A new megaraptoran dinosaur from the Lower Cretaceous Wessex Formation, UK, and its implications for theropod evolution and biogeography
Source: R Soc Open Sci. 2026 Oct 14;13(10):260937. doi: 10.1098/rsos.260937 (PMC13682568; doi:10.1098/rsos.260937)
Supplement: Supplementary file 1 [file rsos.260937.zip › rsos.260937/rsos260937_si_001.pdf]

**Supplementary Information for: 'A new megaraptoran dinosaur from the Lower Cretaceous Wessex Formation, United Kingdom, and its implications for theropod evolution and biogeography'**

Contents


1) Character changes and scoring
2) Supplementary Figures
3) Stratigraphical log


# 1) Character changes and scoring

- Using the Matrix and scoring from Naish & Cau, 2022 (see their supplementary file for the full character list)
- Characters 112 and 632 were revised (see Supplementary Information for new scores) and a new character (1760) was added:

**Amended characters:**

112. Foramen magnum, shape: subcircular, with approximately equal dorsoventral height to mediolateral width (0); elliptical, taller dorsoventrally than mediolaterally wide (1); elliptical, wider mediolaterally than dorsoventrally high (2). Unordered.

- Previously: 112): Foramen magnum, shape: subcircular (0); elliptical, taller than wide (1).

632. Occipital condyle, shape: subcircular, with approximately equal dorsoventral height to mediolateral width (0); elliptical, mediolaterally broader than dorsoventrally tall (1).

- *Spinosaurus* was rescored from a ? to a {0}
- Previously: 112): Foramen magnum, shape: subcircular (0); elliptical, taller than wide (1).

**New character**

1760. Foramen magnum, mediolateral width to that of occipital condyle ratio (FOR): 0.50 or lower (0); 0.51–0.64 (1); 0.65 to 0.95 (2); greater than 0.95 (3). Unordered.

- scoring of all applicable taxa is in the scored character matrix

## 2) Supplementary Figures 1 and 2: Phylogenetic analysis of *Invictibellatrix amberina* in the Ibiricu et al. (2025) matrix.

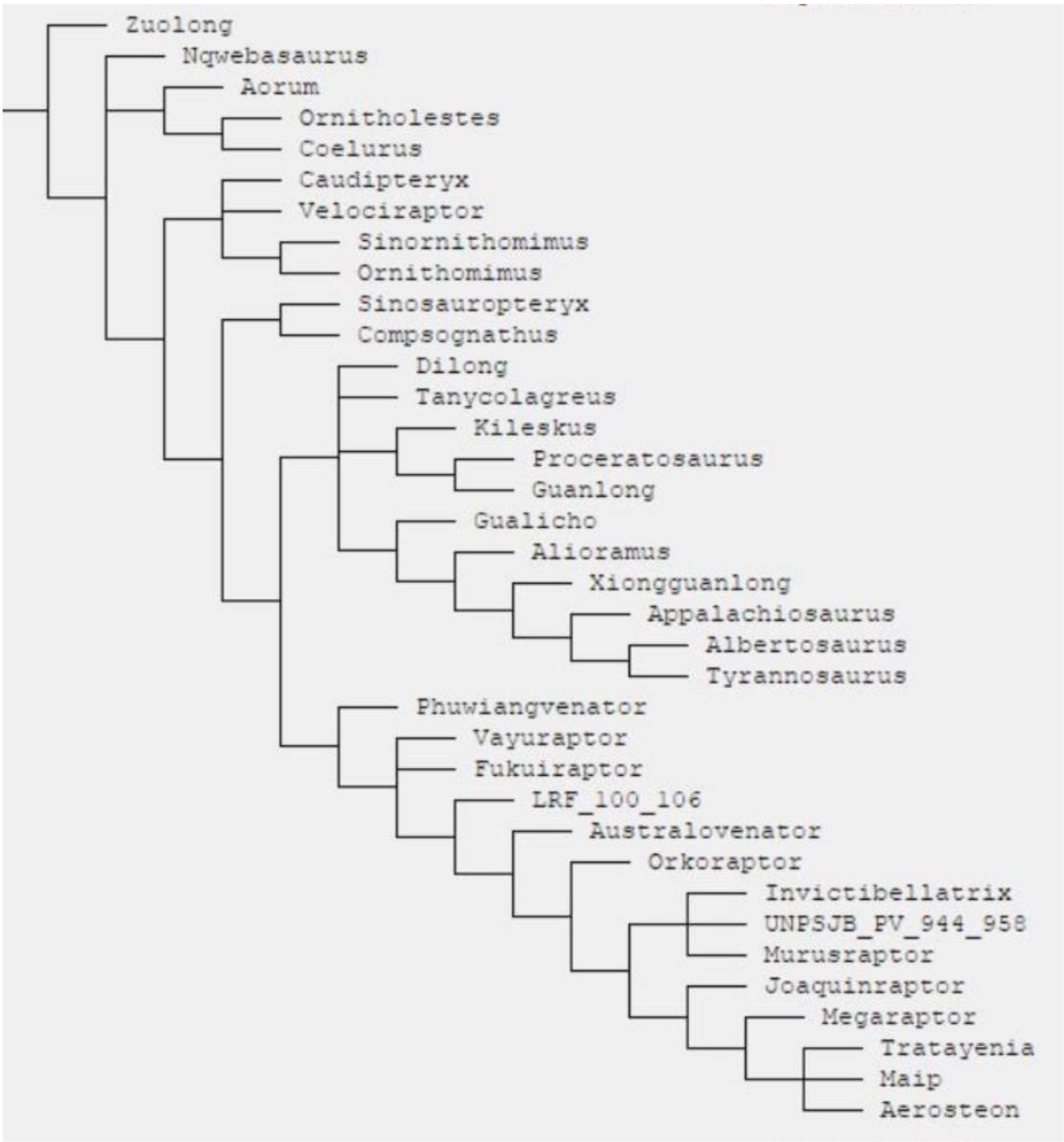


Figure S1: Strict consensus topology of the phylogenetic analysis based on the Ibiricu et al. (2025) data matrix under equal weights. The tree is focused on the results for Coelurosauria. *Invictibellatrix* is nested within Megaraptora.

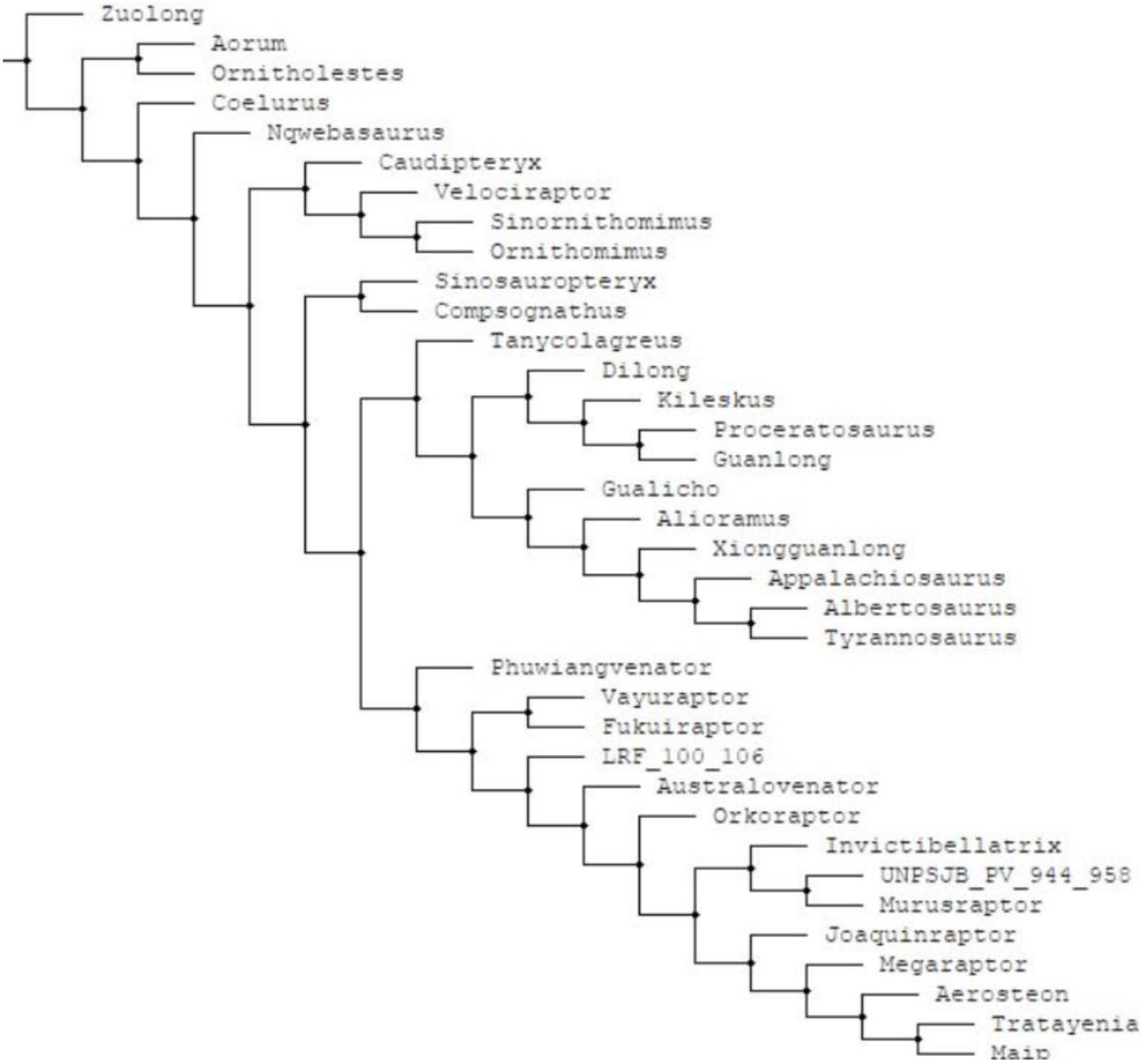


Figure S2: Strict consensus topology of the phylogenetic analysis based on the Ibiricu et al. (2025) data matrix under extended implied weights (K = 9). The tree is focused on the results for Coelurosauria. *Invictibellatrix* is nested within Megaraptora.

# 3) Stratigraphical log

The stratigraphic log contains clades and genera that met the following criteria below:

- Must be published in scientific literature, and/or available for study in public collections, at the time of this publication
- The general clade level assignation for each specimen must be discernible, e.g. no heavily worn / non-diagnostic dinosaur material included
- The general location of each specimen must be known, with bed specific locality data preferred. e.g. no “Isle of Wight” or “Brighstone bay”, as these are large areas where stratigraphic position would be very difficult to determine.

<u>THEROPODA</u>:

*Aristosuchus:*

- General location based on information from NHMUK collections

*Eotyrannus:*

- Naish, D.; Cau, A. (July 2022). "The osteology and affinities of *Eotyrannus lengi*, a tyrannosauroid theropod from the Wealden Supergroup of southern England". PeerJ. 10: e12727. doi:10.7717/peerj.12727. PMC 9271276. PMID 35821895.

*Neovenator:*

- Brusatte, S. L. and Benson, R. B. J. and Hutt, S. (2008) The osteology of *Neovenator salerii* (Dinosauria: Theropoda) from the Wealden Group (Barremian) of the Isle of Wight. Other. Palaeontographical Society, Palaeontographical Society Monographs 162 (631).

*Ceratosuchops* and *Riparovenator*:

- Barker, C.T.; Hone, D.; Naish, D.; Cau, A.; Lockwood, J.; Foster, B.; Clarkin, C.; Schneider, P.; Gostling, N. (2021). "New spinosaurids from the Wessex Formation (Early Cretaceous, UK) and the European origins of Spinosauridae". Scientific Reports. 11 (1): 19340. Bibcode:2021NatSR..1119340B. doi:10.1038/s41598-021-97870-8. PMC 8481559. PMID 34588472.

*Vectiraptor*:

- Longrich, Nicholas R.; Martill, David M.; Jacobs, Megan L. (17 December 2021). "A new dromaeosaurid dinosaur from the Wessex Formation (Lower Cretaceous, Barremian) of the Isle of Wight, and implications for European palaeobiogeography". Cretaceous Research. 134: 105123. doi:10.1016/j.cretres.2021.105123. ISSN 0195-6671. S2CID 245324247.

Dromaeosauridae (excluding *Vectiraptor*):

- Sweetman, S.C., 2004. "The first record of velociraptorine dinosaurs (Saurischia, Theropoda) from the Wealden (Early Cretaceous, Barremian) of southern England". *Cretaceous Research* **25**: 353-364

Tetanurae indet. (MIWG 6350)

- Benson, R. B., Brusatte, S. L., Hutt, S., & Naish, D. (2009). A new large basal tetanuran (Dinosauria: Theropoda) from the Wessex Formation (Barremian) of the Isle of Wight, England. *Journal of vertebrate Paleontology*, *29*(2), 612-615.

*Thecocoelurus*:

- General location based on information from NHMUK collections

Troodontidae:

- Sweetman, S. C. (2007). Aspects of the microvertebrate fauna of the Early Cretaceous (Barremian) Wessex Formation of the Isle of Wight, southern England (Doctoral dissertation, University of Portsmouth).

Other misc. theropods not yet cited:

- Sweetman, S. C. (2007). Aspects of the microvertebrate fauna of the Early Cretaceous (Barremian) Wessex Formation of the Isle of Wight, southern England (Doctoral dissertation, University of Portsmouth).

- Various theropod remains based on information from NHMUK collections and recorded affinities

<u>ORNITHISCHIA:</u>

ANKYLOSAURIA

- Blows, W. T. (2015). British polacanthid dinosaurs: Observations on the history and palaeontology of the UK polacanthid armoured dinosaurs and their relatives. Siri Scientific Press.

*Polacanthus*:

- Pond, S., Strachan, S. J., Raven, T. J., Simpson, M. I., Morgan, K., & Maidment, S. C. (2023). *Vectipelta barretti*, a new ankylosaurian dinosaur from the Lower Cretaceous Wessex Formation of the Isle of Wight, UK. *Journal of Systematic Palaeontology*, *21*(1), 2210577.

*Vectipelta*:

- Pond, S., Strachan, S. J., Raven, T. J., Simpson, M. I., Morgan, K., & Maidment, S. C. (2023). *Vectipelta barretti*, a new ankylosaurian dinosaur from the Lower Cretaceous Wessex Formation of the Isle of Wight, UK. *Journal of Systematic Palaeontology*, *21*(1), 2210577.

DRYOSAURIDAE

- Barrett, P. M., Butler, R. J., Twitchett, R. J., & Hutt, S. (2011). New material of *Valdosaurus canaliculatus* (Ornithischia: Ornithopoda) from the Lower Cretaceous of southern England. In *Studies on Fossil Tetrapods* (pp. 131-163). The Palaeontological Association.

- Barrett, P.M., 2016. A new specimen of *'Valdosaurus canaliculatus'*(Ornithopoda: Dryosauridae) from the Lower Cretaceous of the Isle of Wight, England. *Memoirs of Museum Victoria*, *74*, pp.29-48.

HYPSILOPHODONTIDAE

*Hypsilophodon*

- Longrich, N. R., Martill, D. M., Munt, M., Green, M., Penn, M., & Smith, S. (2024). *Vectidromeus insularis*, a new hypsilophodontid dinosaur from the Lower Cretaceous Wessex Formation of the Isle of Wight, England. Cretaceous Research, 154, 105707.

*Vectidromeus*

- Longrich, N. R., Martill, D. M., Munt, M., Green, M., Penn, M., & Smith, S. (2024). *Vectidromeus insularis*, a new hypsilophodontid dinosaur from the Lower Cretaceous Wessex Formation of the Isle of Wight, England. *Cretaceous Research*, *154*, 105707.

IGUANODONTS

*Brighstoneus*:

- Lockwood, J.A., Martill, D.M. and Maidment, S.C., 2021. A new hadrosauriform dinosaur from the Wessex Formation, Wealden Group (Early Cretaceous), of the Isle of Wight, southern England. Journal of Systematic Palaeontology, 19(12), pp.847-888.

*Comptonatus*:

- Lockwood, J. A., Martill, D. M., & Maidment, S. C. (2024). *Comptonatus chasei*, a new iguanodontian dinosaur from the Lower Cretaceous Wessex Formation of the Isle of Wight, southern England. *Journal of Systematic Palaeontology*, *22*(1), 2346573.

Other ornithischians not yet cited:

- Sweetman, S. C. (2007). Aspects of the microvertebrate fauna of the Early Cretaceous (Barremian) Wessex Formation of the Isle of Wight, southern England (Doctoral dissertation, University of Portsmouth).

SAUROPODA

- Upchurch, P., Mannion, P. D., & Barrett, P. M. (2011). Sauropod dinosaurs. Field Guide t, 1.
- Mannion, P. D., Upchurch, P., & Hutt, S. (2011). New rebbachisaurid (Dinosauria: Sauropoda) material from the Wessex Formation (Barremian, Early Cretaceous), Isle of Wight, United Kingdom. *Cretaceous Research*, *32*(6), 774-780.
