# Supplementary material for: A new megaraptoran dinosaur from the Lower Cretaceous Wessex Formation, UK, and its implications for theropod evolution and biogeography
Source: R Soc Open Sci. 2026 Oct 14;13(10):260937. doi: 10.1098/rsos.260937 (PMC13682568; doi:10.1098/rsos.260937)

BioGeoBEARS BAYAREALIKE on data M0_unconstrained
ancstates: global optim, 3 areas max. d=0.051; e=0.0449; j=0; LnL=−168.23

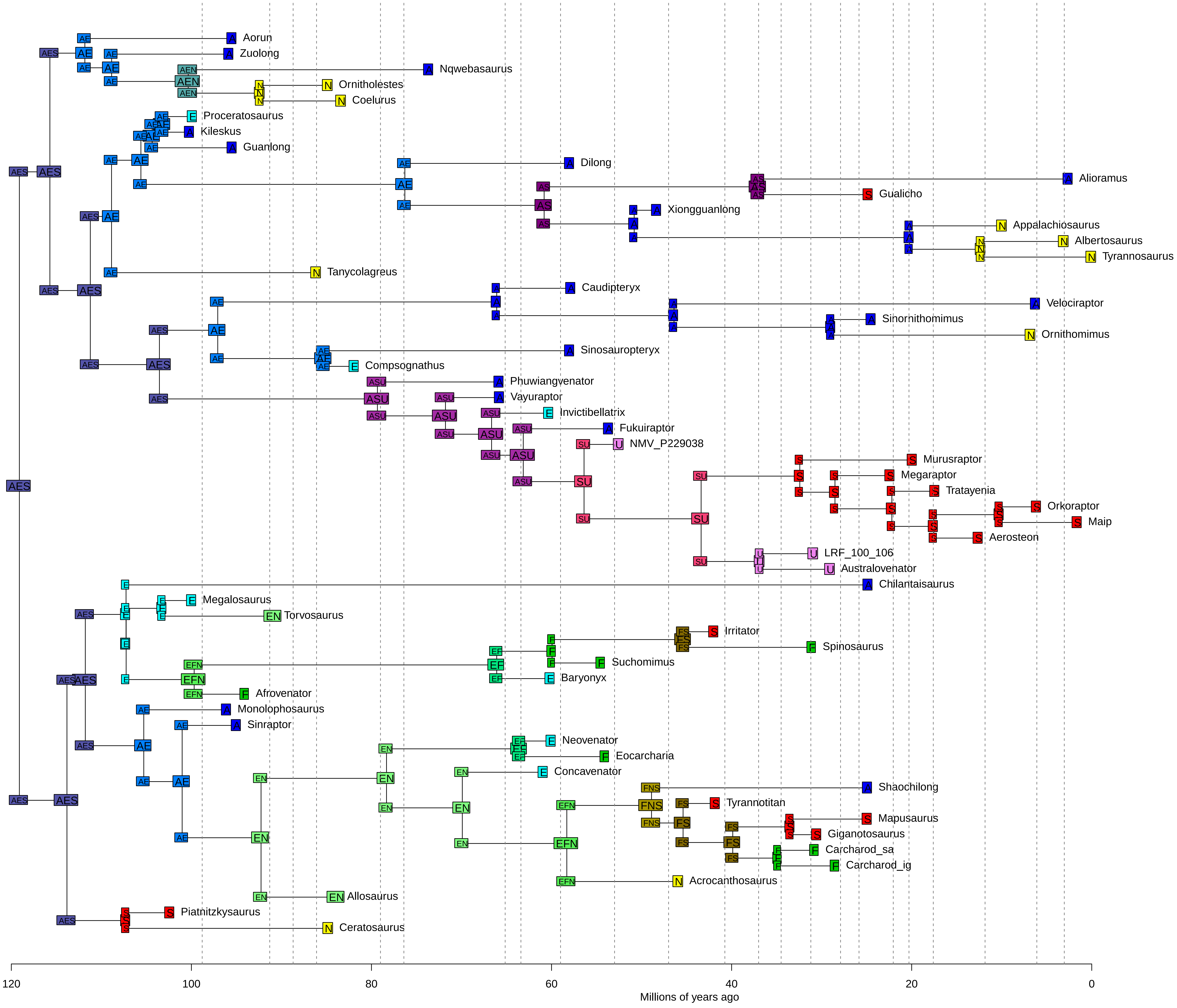

BioGeoBEARS BAYAREALIKE on data M0_unconstrained
ancstates: global optim, 3 areas max. d=0.051; e=0.0449; j=0; LnL=−168.23

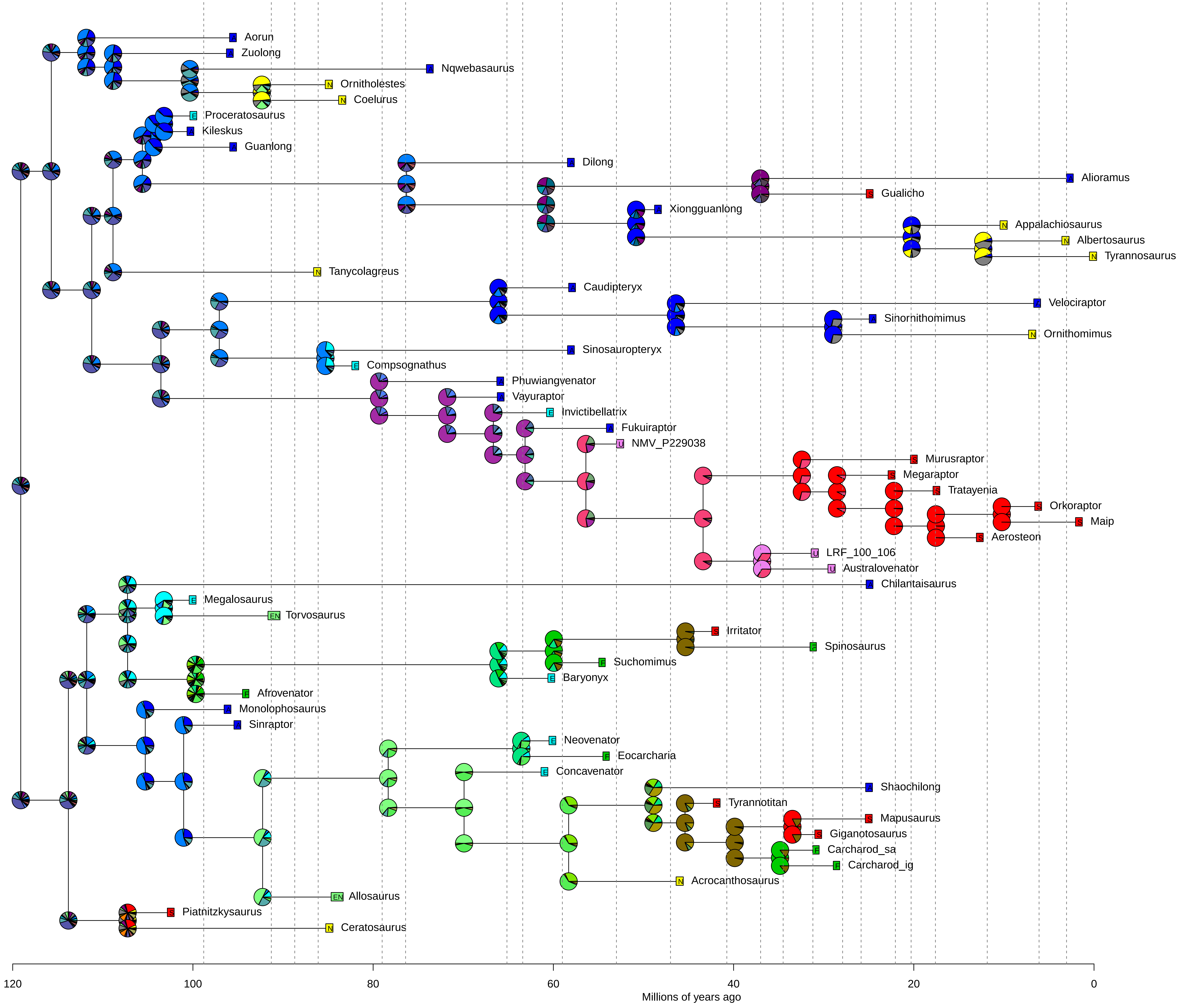

BioGeoBEARS BAYAREALIKE+J on data M0_unconstrained
ancstates: global optim, 3 areas max. d=0.0303; e=0.0302; j=0.2461; LnL=−159.28

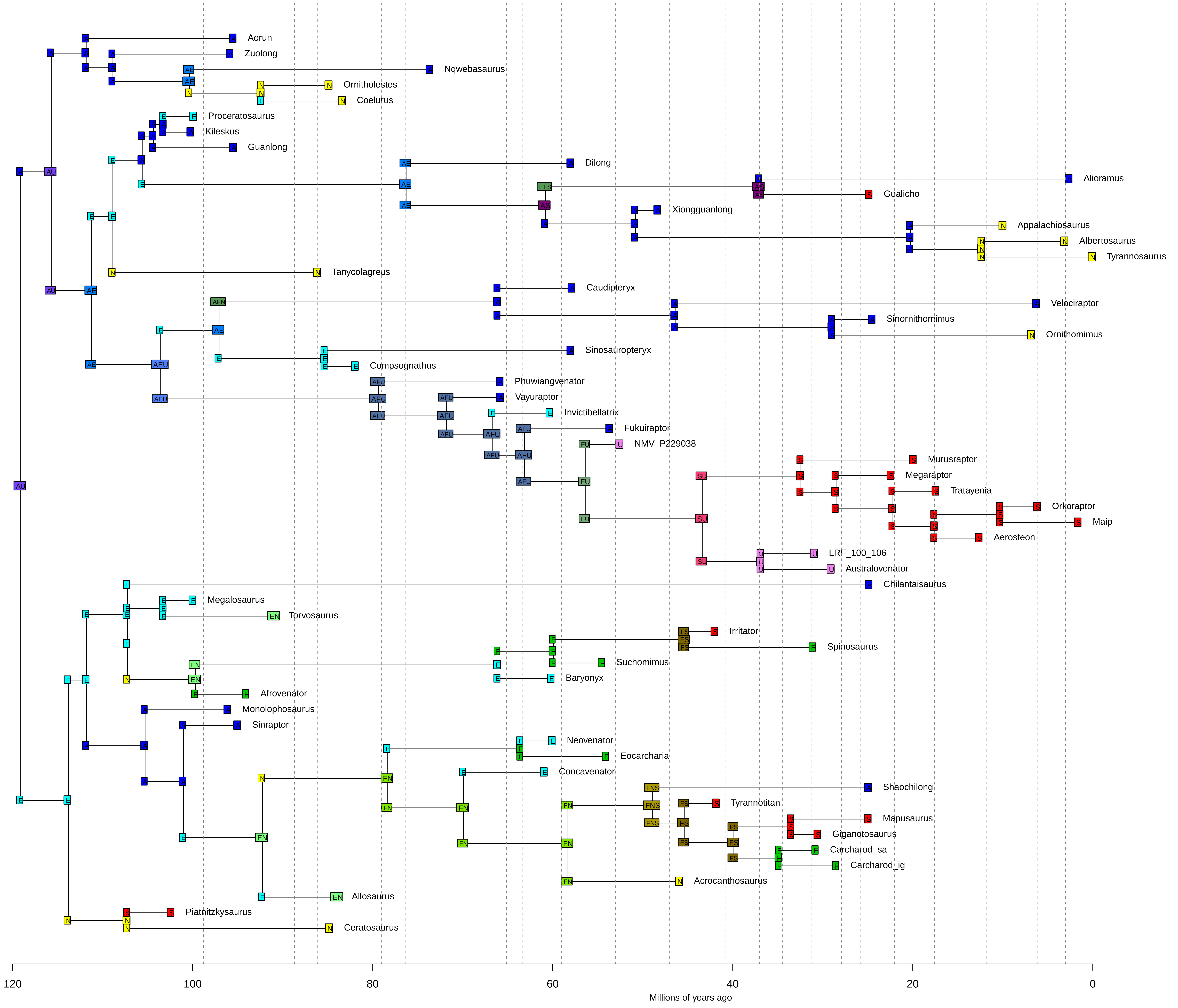

BioGeoBEARS BAYAREALIKE+J on data M0_unconstrained
ancstates: global optim, 3 areas max. d=0.0303; e=0.0302; j=0.2461; LnL=−159.28

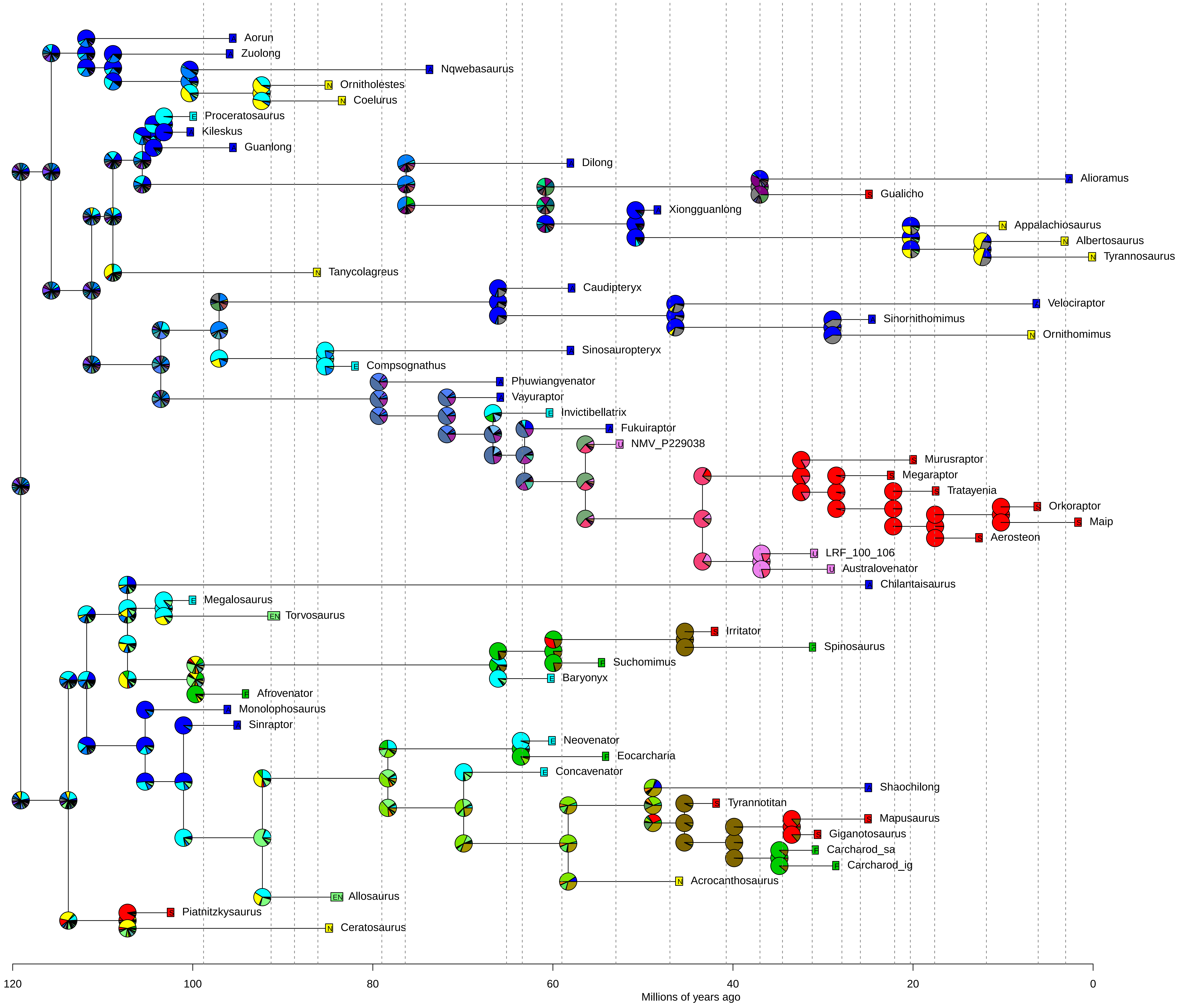

Supplement: Supplementary file 1 [file rsos.260937.zip › rsos.260937/rsos260937_si_002.zip!/biogeography/NEW BIOGEOGRAPHY/NEW BIOGEOGRAPHY/New harsh/BAYAREALIKE_vs_BAYAREALIKE+J_Theropoda_v1.pdf]
