# Supplementary material for: A new megaraptoran dinosaur from the Lower Cretaceous Wessex Formation, UK, and its implications for theropod evolution and biogeography
Source: R Soc Open Sci. 2026 Oct 14;13(10):260937. doi: 10.1098/rsos.260937 (PMC13682568; doi:10.1098/rsos.260937)

BioGeoBEARS DEC on data M0
ancstates: global optim, 3 areas max. d=0.0446; e=0.0111; j=0; LnL=−141.05

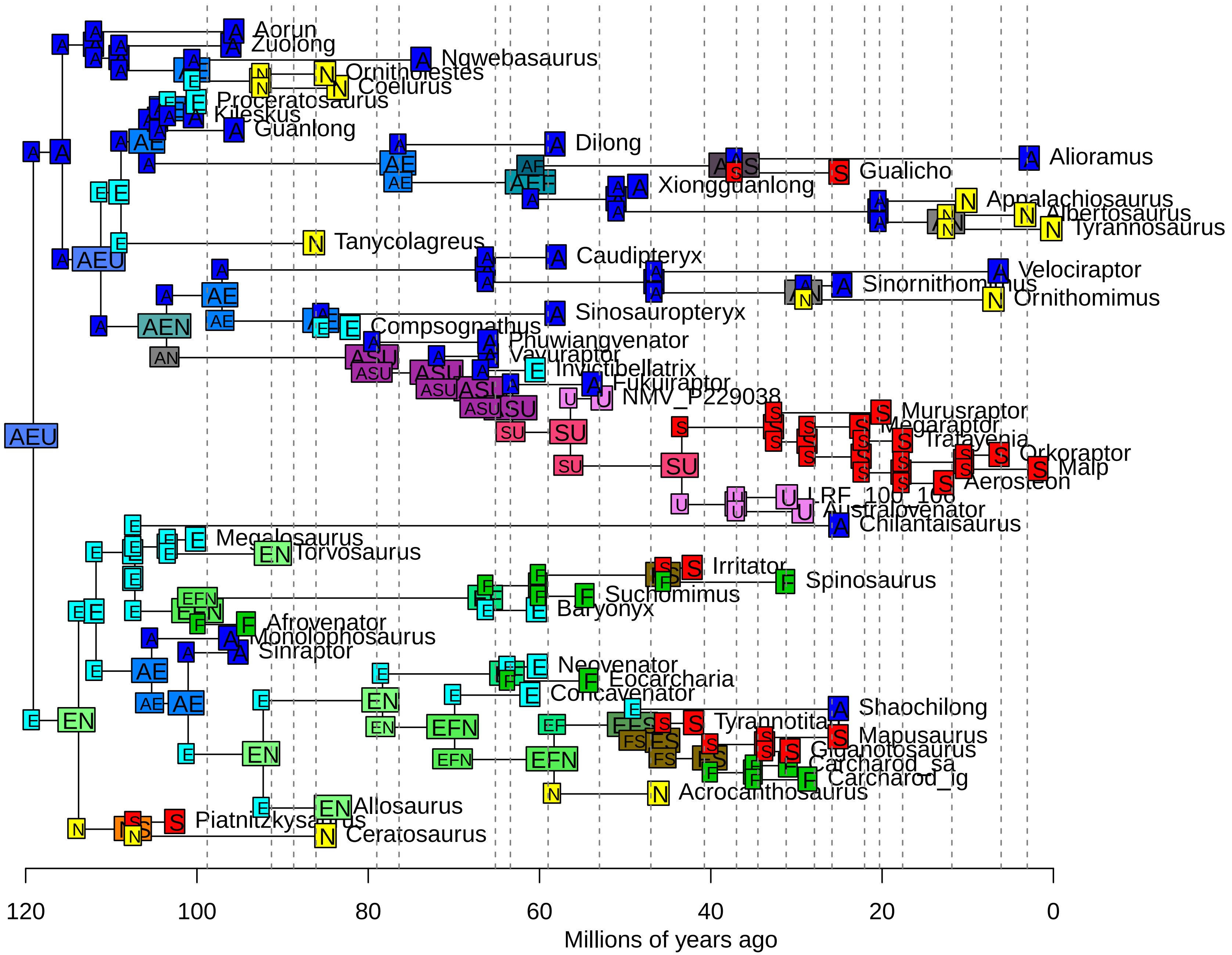

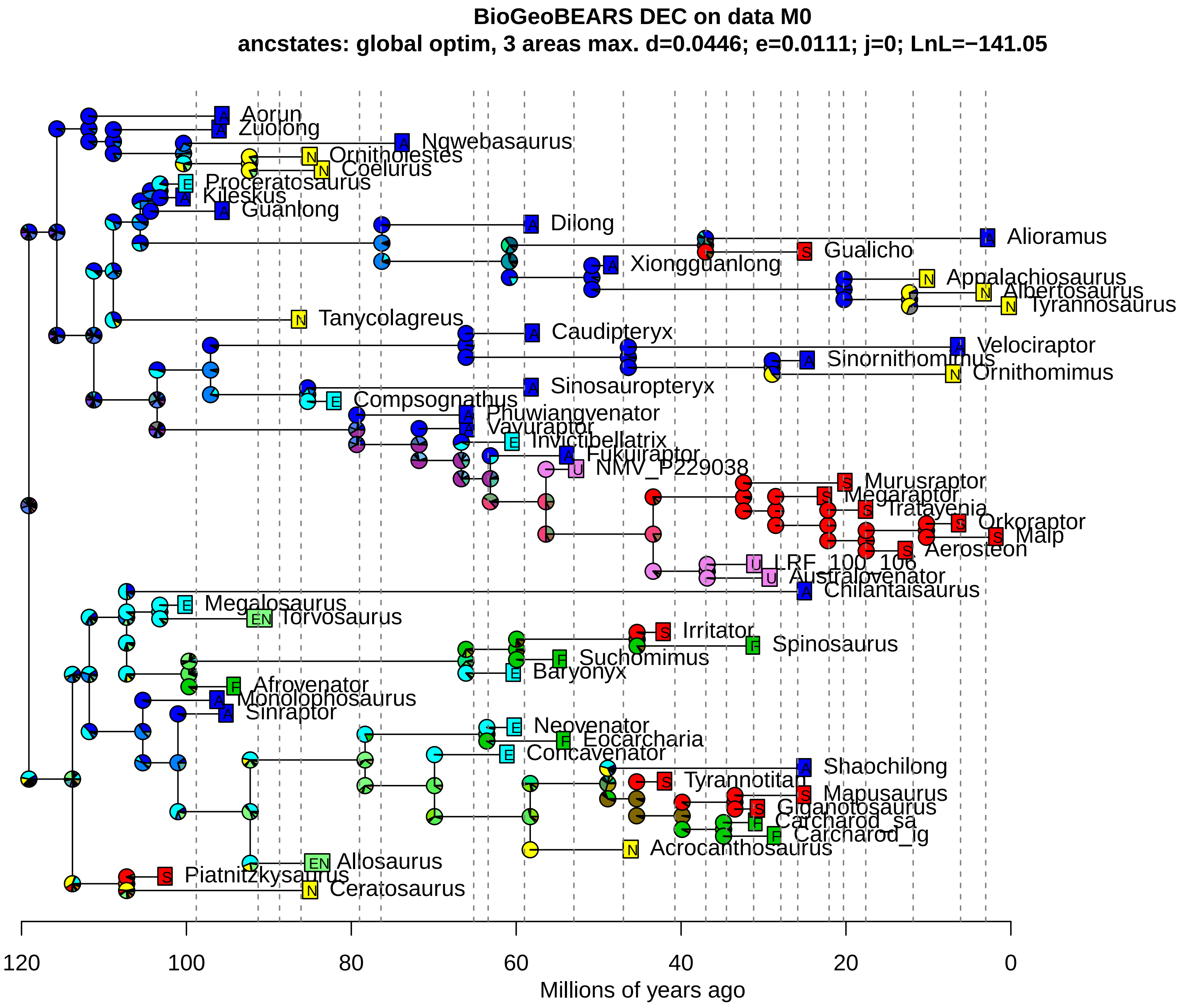

BioGeoBEARS DEC on data M0
ancstates: global optim, 3 areas max. d=0.0446; e=0.0111; j=0; LnL=−141.05
Aorun
Zuolong
Nqwebasaurus
Ornitholestes
Coelurus
Proceratosaurus
Kileskus
Guanlong
Dilong
Alioramus
Gualicho
Xiongguanlong
Appalachiosaurus
Albertosaurus
Tyrannosaurus
Tanycolagreus
Caudipteryx
Velociraptor
Sinornithomimus
Ornithomimus
Sinosauropteryx
Compsognathus
Phuwiangvenator
Vayuraptor
Invictibellatrix
Fukuiraptor
NMV_P229038
Murusraptor
Megaraptor
Tratayenia
Orkoraptor
Malp
Aerosteon
LRF_100_106
Australovenator
Chilantaisaurus
Megalosaurus
Torvosaurus
Irritator
Spinosaurus
Suchomimus
Baryonyx
Afrovenator
Monolophosaurus
Sinraptor
Neovenator
Eocarcharia
Concavenator
Shaochilong
Tyrannotitan
Mapusaurus
Giganotosaurus
Carcharod_sa
Carcharod_ig
Acrocanthosaurus
Allosaurus
Piatnitzkysaurus
Ceratosaurus
120
100
80
60
40
20
0
Millions of years ago

BioGeoBEARS DEC+J
ancstates: global optim, 3 areas max. d=0.0257; e=0.0071; j=0.6175; LnL=−132.46

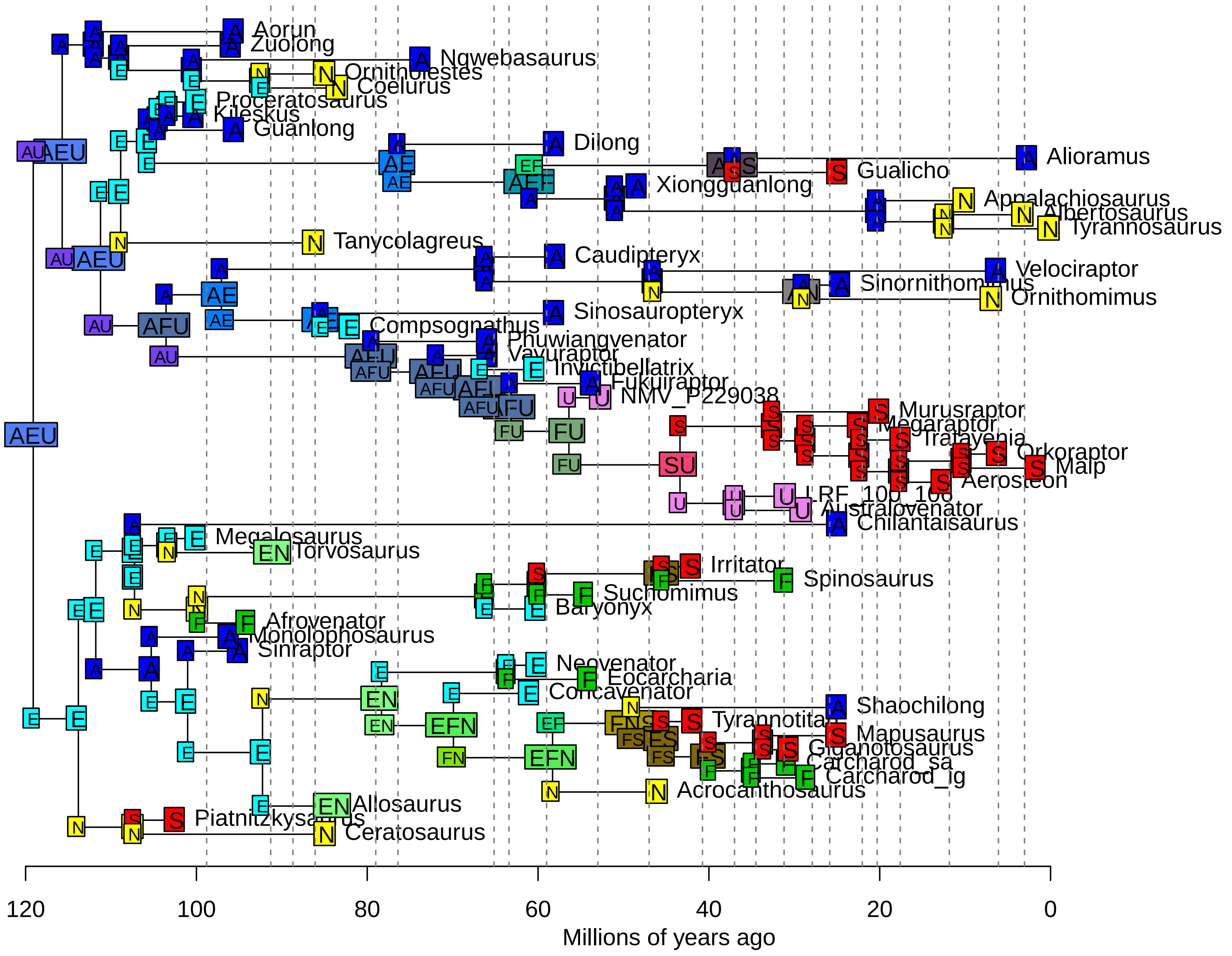

BioGeoBEARS DEC+J

ancstates: global optim, 3 areas max. d=0.0257; e=0.0071; j=0.6175; LnL=−132.46

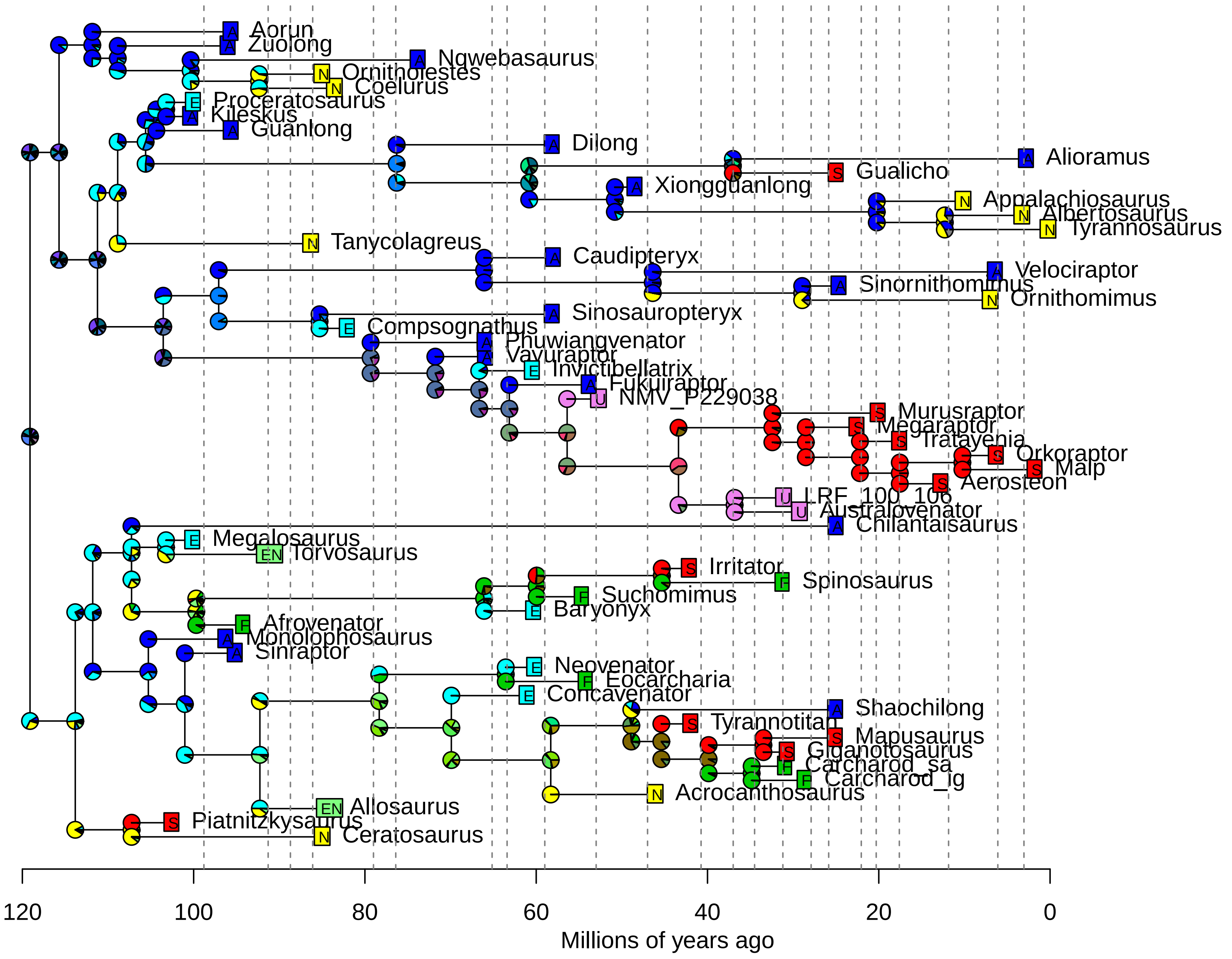

Supplement: Supplementary file 1 [file rsos.260937.zip › rsos.260937/rsos260937_si_002.zip!/biogeography/NEW BIOGEOGRAPHY/NEW BIOGEOGRAPHY/New harsh/Tyrannosauroidea Phylogeny.pdf]
