# Supplementary material for: A new megaraptoran dinosaur from the Lower Cretaceous Wessex Formation, UK, and its implications for theropod evolution and biogeography
Source: R Soc Open Sci. 2026 Oct 14;13(10):260937. doi: 10.1098/rsos.260937 (PMC13682568; doi:10.1098/rsos.260937)

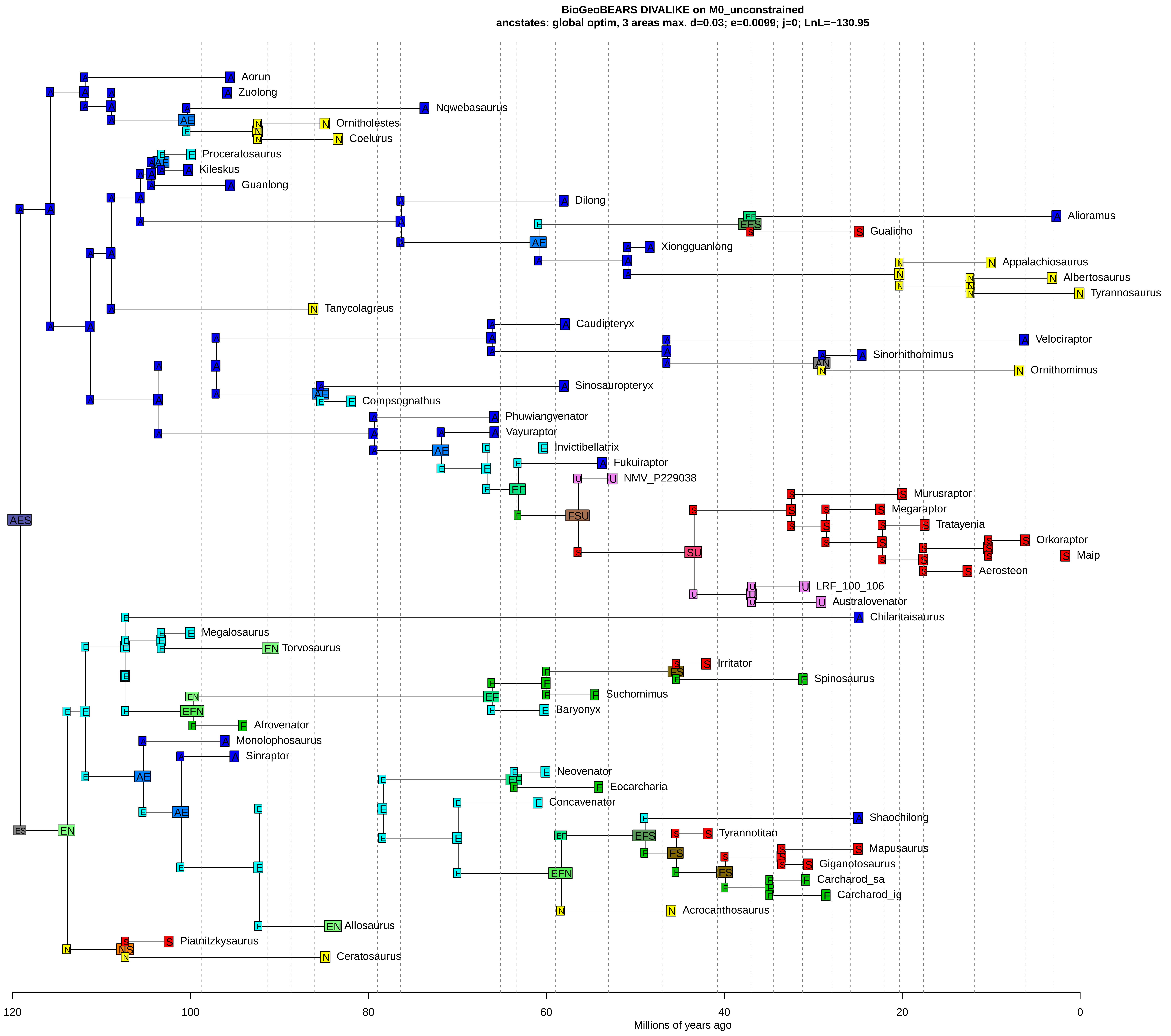

BioGeoBEARS DIVALIKE on M0_unconstrained
ancstates: global optim, 3 areas max. d=0.03; e=0.0099; j=0; LnL=−130.95
Aorun
Zuolong
Nqwebasaurus
Ornitholestes
Coelurus
Proceratosaurus
Kileskus
Guanlong
Dilong
Alioramus
Gualicho
Xiongguanlong
Appalachiosaurus
Albertosaurus
Tyrannosaurus
Tanycolagreus
Caudipteryx
Velociraptor
Sinornithomimus
Ornithomimus
Sinosauropteryx
Compsognathus
Phuwiangvenator
Vayuraptor
Invictibellatrix
Fukuiraptor
NMV_P229038
Murusraptor
Megaraptor
Tratayenia
Orkoraptor
Maip
Aerosteon
LRF_100_106
Australovenator
Chilantaisaurus
Megalosaurus
Torvosaurus
Irritator
Spinosaurus
Suchomimus
Baryonyx
Afrovenator
Monolophosaurus
Sinraptor
Neovenator
Eocarcharia
Concavenator
Shaochilong
Tyrannotitan
Mapusaurus
Giganotosaurus
Carcharod_sa
Carcharod_ig
Acrocanthosaurus
Allosaurus
Piatnitzkysaurus
Ceratosaurus
120
100
80
60
40
20
0
Millions of years ago

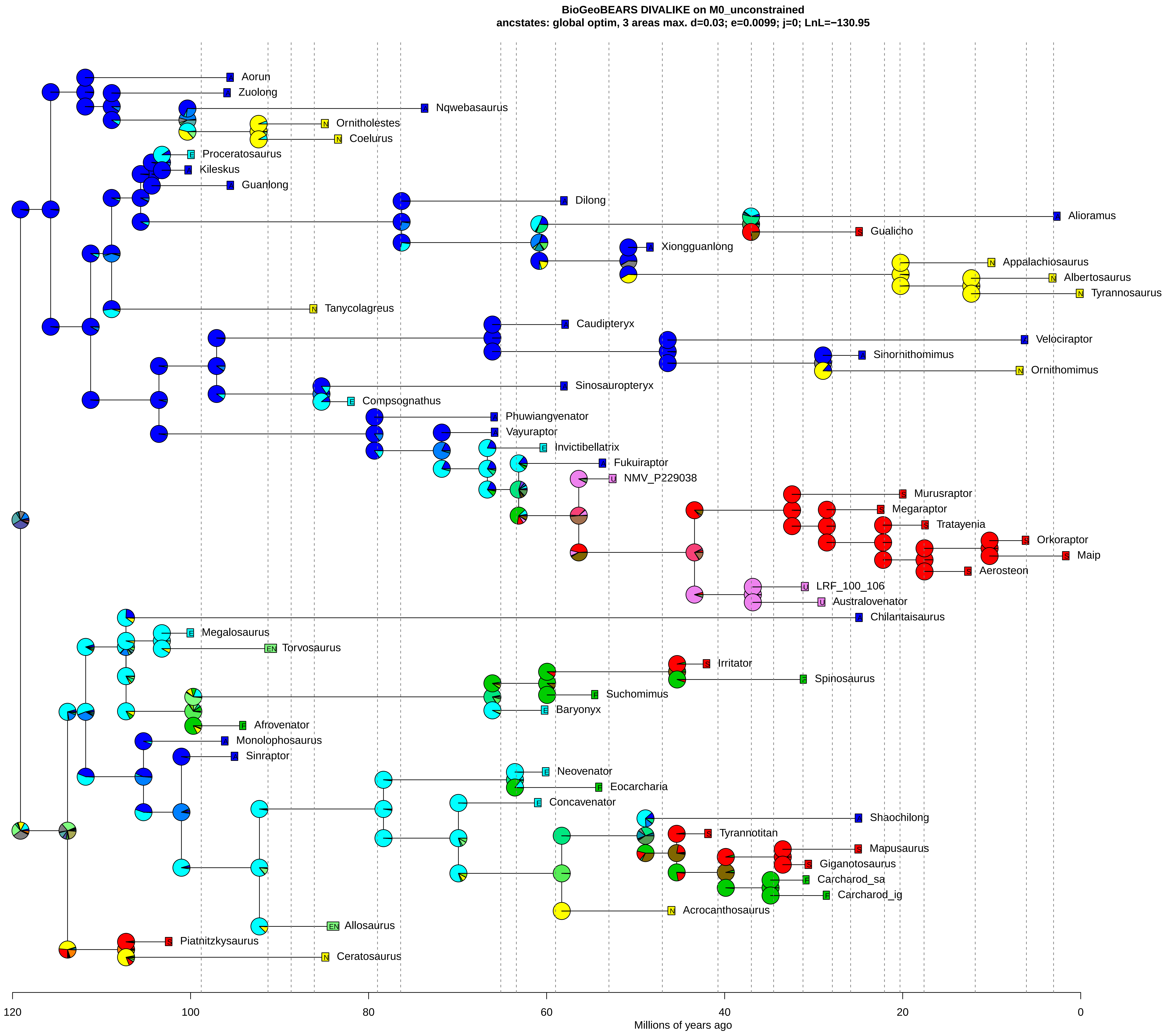

BioGeoBEARS DIVALIKE on M0_unconstrained
ancstates: global optim, 3 areas max. d=0.03; e=0.0099; j=0; LnL=−130.95
Aorun
Zuolong
Nqwebasaurus
Ornitholestes
Coelurus
Proceratosaurus
Kileskus
Guanlong
Dilong
Alioramus
Gualicho
Xiongguanlong
Appalachiosaurus
Albertosaurus
Tyrannosaurus
Tanycolagreus
Caudipteryx
Velociraptor
Sinornithomimus
Ornithomimus
Sinosauropteryx
Compsognathus
Phuwiangvenator
Vayuraptor
Invictibellatrix
Fukuiraptor
NMV_P229038
Murusraptor
Megaraptor
Tratayenia
Orkoraptor
Maip
Aerosteon
LRF_100_106
Australovenator
Chilantaisaurus
Megalosaurus
Torvosaurus
Irritator
Spinosaurus
Suchomimus
Baryonyx
Afrovenator
Monolophosaurus
Sinraptor
Neovenator
Eocarcharia
Concavenator
Shaochilong
Tyrannotitan
Mapusaurus
Giganotosaurus
Carcharod_sa
Carcharod_ig
Acrocanthosaurus
Allosaurus
Piatnitzkysaurus
Ceratosaurus
120
100
80
60
40
20
0
Millions of years ago

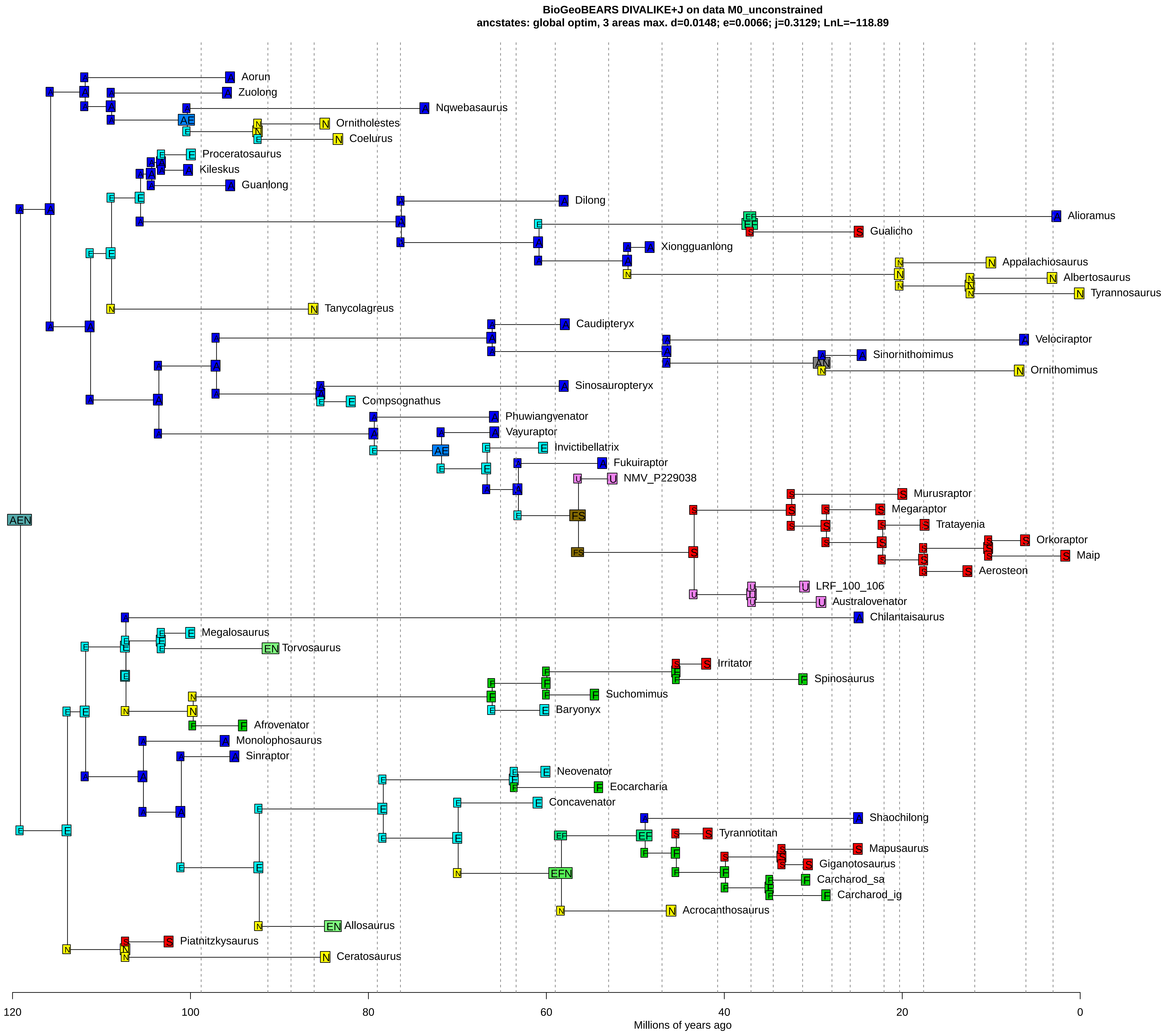
BioGeoBEARS DIVALIKE+J on data M0_unconstrained
ancstates: global optim, 3 areas max. d=0.0148; e=0.0066; j=0.3129; LnL=−118.89
Aorun
Zuolong
Nqwebasaurus
Ornitholestes
Coelurus
Proceratosaurus
Kileskus
Guanlong
Dilong
Alioramus
Gualicho
Xiongguanlong
Appalachiosaurus
Albertosaurus
Tyrannosaurus
Tanycolagreus
Caudipteryx
Velociraptor
Sinornithomimus
Ornithomimus
Sinosauropteryx
Compsognathus
Phuwiangvenator
Vayuraptor
Invictibellatrix
Fukuiraptor
NMV_P229038
Murusraptor
Megaraptor
Tratayenia
Orkoraptor
Maip
Aerosteon
LRF_100_106
Australovenator
Chilantaisaurus
Megalosaurus
Torvosaurus
Irritator
Spinosaurus
Suchomimus
Baryonyx
Afrovenator
Monolophosaurus
Sinraptor
Neovenator
Eocarcharia
Concavenator
Shaochilong
Tyrannotitan
Mapusaurus
Giganotosaurus
Carcharod_sa
Carcharod_ig
Acrocanthosaurus
Allosaurus
Piatnitzkysaurus
Ceratosaurus
AEN
120
100
80
60
40
20
0
Millions of years ago

BioGeoBEARS DIVALIKE+J on data M0_unconstrained
ancstates: global optim, 3 areas max. d=0.0148; e=0.0066; j=0.3129; LnL=−118.89

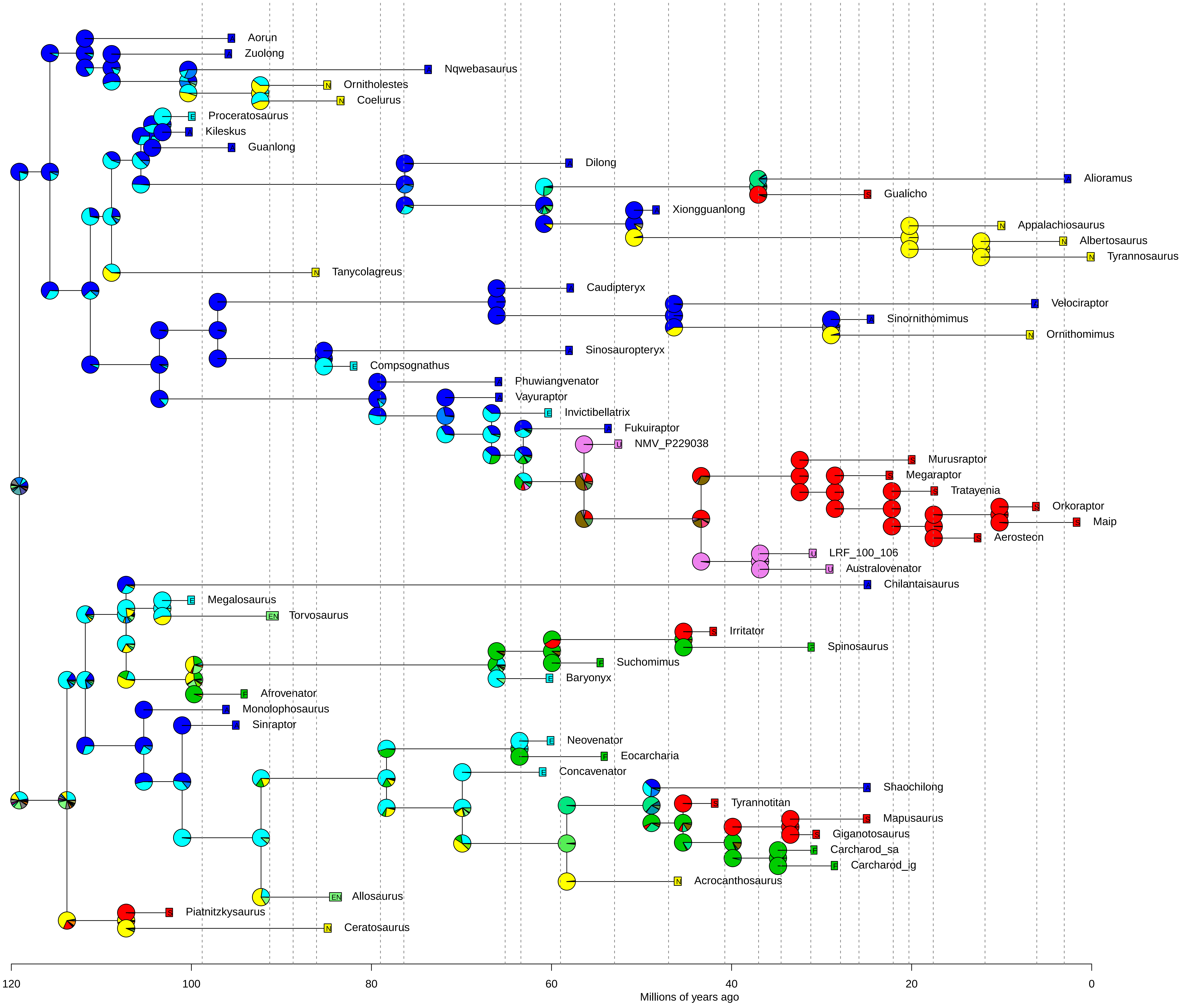

Supplement: Supplementary file 1 [file rsos.260937.zip › rsos.260937/rsos260937_si_002.zip!/biogeography/NEW BIOGEOGRAPHY/NEW BIOGEOGRAPHY/New relaxed/DIVALIKE_vs_DIVALIKE+J_Theropoda_v1.pdf]
